# γ-aminobutyric acid is associated with calcium, reactive oxygen species, and nitric oxide responses and enhanced cadmium tolerance in *Salvadora persica*

**DOI:** 10.1080/15592324.2026.2748062

**Published:** 2026-10-14

**Authors:** Shazia Perveen, Imran Haider, Azizullah Khalili, Mohamed M. El-Mogy, Husameldin H. Mahmoud, Tarek A. Shalaby

**Affiliations:** a Department of Zoology, The Women University Multan, Multan, Pakistan; b National Research Center of Intercropping, The Islamia University of Bahawalpur, Bahawalpur, Pakistan; c Department of Biology, Nakhchivan State University, Nakhchivan Azerbaijan; d Department of Agronomy, Faculty of Agriculture, Sayed Jamaluddin Afghani University (SJAU), Kunar, Afghanistan; e Department of Arid Land Agriculture, College of Agricultural and Food Sciences, King Faisal University, Al-Ahsa, Saudi Arabia

**Keywords:** Cadmium, calcium-sensitive fluorescence, γ-aminobutyric acid, medicinal plant, nitric oxide-sensitive fluorescence, phytochelatins, reactive oxygen species, *Salvadora persica*

## Abstract

**Background:**

*γ*-Aminobutyric acid (GABA) accumulates under stress, but its association with calcium (Ca^2+^), reactive oxygen species (ROS), nitric oxide (NO), and thiol responses during cadmium (Cd) exposure remains unclear in medicinal shrubs.

**Methods:**

Rooted *Salvadora persica* L. cuttings were grown in sand-soil substrate. Screening selected 50mg Cd kg^−1^ substrate and 0.5  mM root-applied GABA. Eleven treatments had five independent pots each. Ca^2+^-sensitive Fluo-3 AM and NO-associated DAF-FM DA fluorescence and H₂O₂ were assessed in separate, destructively sampled plants at five post-application times. The 0-min value was a post-application reference; probe fluorescence was not calibrated to analyte concentration. Pot means were independent units, with time treated as a between-pot factor. A core Cd × GABA × time model and a separate categorical endpoint model were specified. The exact *p*-values, adjusted q-values, and model diagnostics could not be verified without pot-level records. Correlation and principal component analyses were exploratory; no path model was fitted.

**Results:**

Cd reduced shoot dry weight from 3.04 to 1.89 g plant^−1^ (37.8%) and increased malondialdehyde from 4.37 to 9.86 nmol g^−1^ FW (125.6%). Relative to Cd alone, GABA increased the shoot dry weight to 2.62 g plant^−1^ (38.6%) and reduced the malondialdehyde to 4.34 nmol g^−1^ FW (56.0%). GABA was associated with a 2.2-fold greater Ca^2+^-sensitive fluorescence response amplitude at 30 min and 2.7-fold greater NO-associated amplitude at 360 min. LaCl₃, EGTA, DPI, DMTU, and cPTIO attenuated GABA-associated protection. Inhibitor-only and rescue controls were absent, and inhibitor phytotoxicity pre-screening was undocumented. GABA also increased total phenolics from 22.77 to 26.54  mg GAE g^−1^ DW (16.6%) and flavonoids from 13.21 to 15.78  mg QE g^−1^ DW (19.5%) relative to Cd alone.

**Conclusion:**

GABA was associated with improved Cd tolerance and differences in sampled fluorescence, H₂O₂, and thiol responses. These data cannot establish a continuous signaling sequence, upstream GABA action, or target-specific pharmacological effects. The attenuation is compatible with network involvement and feedback, but pathway direction and pharmacological specificity require inhibitor-only controls, rescue experiments, and independent, well-controlled temporal validation in future studies.

## 1. Introduction

*γ*-Aminobutyric acid (GABA) is a four-carbon non-protein amino acid that functions as a metabolic intermediate and stress signal. Recent evidence has shown that GABA regulates anion transport, the membrane potential, stomatal behavior, respiratory metabolism, antioxidant capacity, osmotic adjustment, and ion homeostasis under environmental stress^1-8^. Its signaling role is especially relevant to metal toxicity because early Ca^2+^ and redox responses may accompany protection, although their timing alone cannot establish whether GABA initiates them^9-12^. Calcium (Ca^2+^) is one of the first messengers activated after stress perception. The amplitude, duration, and spatial distribution of cytosolic Ca^2+^ form stimulus-specific signatures that are decoded by Ca^2+^-binding proteins and protein kinases.^13-15^ These decoders activate respiratory burst oxidase homologs at the plasma membrane, connecting Ca^2+^ signals with reactive oxygen species (ROS) production and rapid root-to-shoot communication.^16-18^ M​easurements at defined times are required to assess whether GABA treatment is associated with altered Ca^2+^-sensitive fluorescence during acclimation.

ROS and nitric oxide (NO) are interconnected components of stress responses. A transient ROS burst serves as a second messenger, whereas prolonged ROS accumulation causes lipid peroxidation, electrolyte leakage, pigment loss, and photosynthetic inhibition.^19-21^ NO interacts with Ca^2+^ and ROS through redox-dependent protein modifications and regulates antioxidant enzymes, stomatal behavior, cell-wall properties, and metal transport.^22-27^ GABA-associated protection may coincide with altered ROS and NO responses and reduced sustained oxidative injury. Glutathione (GSH) and phytochelatins are detoxification metabolites whose concentrations can also change during Cd exposure. Cd displaces essential metals, binds sulfhydryl groups, disrupts chloroplast and mitochondrial electron transport, and depletes cellular GSH, thereby intensifying oxidative injury.^28-32^ Plants counter this toxicity through antioxidant defense and by converting GSH into phytochelatins, which chelate Cd and facilitate its vacuolar sequestration.^33-35^ If Ca^2+^, ROS, and NO processes contribute to the GABA-associated response, perturbing these processes may attenuate thiol accumulation and the observed physiological protection; chemical perturbation alone cannot establish their order or specific molecular targets.

*Salvadora persica* L. is a stress-tolerant medicinal shrub valued for oral health applications and bioactive constituents, including benzyl isothiocyanate, phenolics, and flavonoids.^36-39^ Its capacity to grow in marginal soils makes Cd tolerance relevant; however, survival alone is insufficient if contamination compromises medicinal quality or product safety.^40-43^ Whether GABA-associated Cd tolerance coincides with Ca^2+^, ROS, NO, GSH, and phytochelatin responses in *S. persica* remains unresolved. We hypothesized that GABA-associated Cd tolerance would coincide with altered Ca^2+^-sensitive and NO-associated relative fluorescence responses, ROS levels, and thiol metabolism. We determined an effective Cd dose and GABA concentration, sampled responses at defined times over 24 h, assessed their pharmacological sensitivity, and compared their Cd partitioning, redox defense, photosynthesis, growth, and measured phenolic and flavonoid concentrations.

Therefore, we evaluated whether exogenous GABA-associated Cd protection coincided with a feedback-linked Ca^2+^-ROS-NO response and downstream thiol, ionic, photosynthetic, phenolic, and flavonoid outcomes. The objectives were (i) to identify a sub-lethal Cd dose and an effective GABA concentration; (ii) to compare Ca^2+^-sensitive and NO-associated relative fluorescence responses and H₂O₂ at five defined times; (iii) to assess responses to LaCl₃, EGTA, DPI, DMTU, and cPTIO; and (iv) to characterize multivariate associations using multiplicity-controlled univariate tests, Pearson correlations, and principal component analysis. No confirmatory path analysis objective was retained because the available design did not support the estimation and reporting of a fully specified path model with coefficients and global fit statistics.

## 2. Materials and methods

### 2.1. Plant material authentication and propagation methods

Semi-hardwood cuttings and mature fruits of *Salvadora persica* L. were collected from naturally growing stands in the Cholistan tract of southern Punjab, Pakistan. A voucher specimen was deposited in the herbarium of the Department of Botany, The Women University Multan, and the identity of the material was confirmed against authenticated herbarium sheets using standard floristic keys. Seed viability was checked before sowing by the tetrazolium test on a random sample of 100 seeds, and only seed lots exceeding 85% viability were used. Because seed-propagated *S. persica* is variable, the main experiment was conducted on uniform-rooted cuttings (12–14 cm, three nodes) raised from a single mother plant, so that genotypic variation between treatments was removed. The cuttings were surface-sterilized in 1% (v/v) sodium hypochlorite for 3 min, rinsed five times in sterile distilled water, and rooted in autoclaved coarse sand under intermittent mist. Uniformly rooted plants were transplanted to experimental pots 21 d after striking and were allowed to establish for a further 14 d before the treatments began. All plants were 35 d old at the start of the treatments.

### 2.2. Growth substrate characterization and growth-condition methods

Plastic pots (18 cm top diameter, 16 cm height) were filled with 3.0 kg of a sand-loam substrate (2:1 v/v) sieved through a 2-mm mesh. The physicochemical properties of the substrate are listed in Table 1. The background Cd concentration was below 0.35  mg kg^−1^, confirming that the substrate was not pre-contaminated and that all measured Cd could be attributed to the applied treatments. Substrate pH and electrical conductivity were determined in a 1:2.5 (w/v) suspension, organic carbon by the Walkley‒Black procedure, total nitrogen by the Kjeldahl method, available phosphorus by the Olsen method, and extractable potassium by ammonium acetate extraction followed by flame photometry. Pseudo-total Cd and Pb were extracted in aqua regia and quantified by atomic absorption spectrophotometry. The experiment was carried out in a controlled-environment glasshouse at the Department of Botany, The Women University Multan (30.20° N, 71.47° E), at 30 ± 2 °C d/22 ± 2 °C night, 60 ± 5% relative humidity, a 14-h photoperiod, and a photosynthetic photon flux density of 650  μmol m^−2^ s at the canopy level supplied by daylight supplemented with LED panels. Pots were watered to 70% of water-holding capacity by weight every second day and re-randomized on the bench weekly to remove positional effects.

**Table 1.** Physicochemical properties of the growth substrate and environmental conditions used in the *Salvadora persica* pot experiment. Values are means ± standard error of five independent composite samples analyzed before the start of the experiment.

Property	Unit	Value
Textural class	–	Sandy loam
pH (1:2.5 H₂O)	–	7.9 ± 0.08
Electrical conductivity	dS m^−1^	1.35 ± 0.04
Organic carbon	%	0.48 ± 0.02
Total nitrogen	%	0.052 ± 0.003
Available phosphorus (Olsen)	mg kg^−1^	7.42 ± 0.21
Extractable potassium	mg kg^−1^	146.3 ± 3.8
Cation exchange capacity	cmolc kg^−1^	8.6 ± 0.3
Background cadmium	mg kg^−1^	0.31 ± 0.02
Background lead	mg kg^−1^	4.18 ± 0.17
Water-holding capacity	%	24.7 ± 0.6
Day/night temperature	°C	30 ± 2/22 ± 2
Relative humidity	%	60 ± 5
Photoperiod	h	14
Photosynthetic photon flux density	μmol m^−2^ s^−1^	650 ± 25
Plant age at treatment	d after striking	35
Pot volume/substrate mass	kg pot^−1^	3.0

Background Cd was well below the applied concentration, confirming that the measured plant Cd originated from the imposed treatments.

### 2.3. Preliminary cadmium dose-screening assay

Cd was supplied as analytical-grade CdCl₂·2.5H₂O at 0, 25, 50, 75, 100, and 150  mg Cd kg. Five replicate pots per dose were evaluated after 21 d (Table 2). This preliminary screen was independent of the subsequent main experiment and had its own controls. At 50  mg Cd kg^-1^, the shoot dry biomass decreased from 3.06 to 2.00 g plant^−1^, a 34.6% reduction, and MDA increased from 4.41 to 9.74 nmol g^−1^ FW, a 120.9% increase; no mortality was observed. Therefore, 50  mg Cd kg^−1^ was selected as the lowest dose that produced substantial but non-lethal injury. The 37.8% shoot dry weight reduction reported for the main experiment was calculated separately from its control and Cd means (3.04 and 1.89 g plant^−1^; Table 3) and does not contradict the 34.6% preliminary-screen value.

**Table 2.** Independent preliminary Cd dose-response screen after 21 d. Values are mean ± standard error (*n* = 5). Percent reduction was calculated within this screening experiment relative to its 0-mg Cd control mean (3.06 g plant^−1^); thus, 50 mg Cd kg^−1^ reduced shoot dry weight to 2.00 g plant^−1^, or by 34.6%.

Cd applied (mg kg^−1^)	Shoot DW (g plant^−1^)	Reduction (%)	F_v_/F_m_	Electrolyte leakage (%)	MDA (nmol g^−1^ FW)	Root Cd (mg kg^−1^)	Shoot Cd (mg kg^−1^)	Mortality (%)
0	3.06 ± 0.07 a	–	0.828 ± 0.004 a	11.9 ± 0.2 f	4.41 ± 0.09 f	0.38 ± 0.02 f	0.15 ± 0.01 f	0
25	2.76 ± 0.06 b	9.8	0.801 ± 0.005 b	19.4 ± 0.4 e	6.28 ± 0.12 e	48.6 ± 1.4 e	21.8 ± 0.7 e	0
50	2.00 ± 0.05 c	34.6	0.726 ± 0.004 c	36.2 ± 0.6 d	9.74 ± 0.20 d	94.7 ± 2.6 d	44.9 ± 1.0 d	0
75	1.58 ± 0.04 d	48.3	0.681 ± 0.006 d	45.8 ± 0.8 c	12.60 ± 0.27 c	141.5 ± 3.9 c	70.3 ± 1.8 c	0
100	1.17 ± 0.04 e	61.7	0.612 ± 0.007 e	57.3 ± 1.1 b	15.82 ± 0.33 b	186.2 ± 4.7 b	97.4 ± 2.4 b	12
150	0.84 ± 0.03 f	72.4	0.534 ± 0.009 f	68.9 ± 1.4 a	19.06 ± 0.41 a	262.8 ± 6.3 a	141.7 ± 3.5 a	28

The screening experiment was separate from the main experiment, as reported in Tables 3 and 6. In the main experiment, the corresponding control and Cd means were 3.04 and 1.89 g plant^−1^, respectively, indicating a 37.8% reduction. The percentages throughout the text were recalculated from the displayed table and rounded to one decimal place.

**Table 3.** Physiological, biochemical, ionic, total phenolic, and total flavonoid traits of *Salvadora persica* at the 24 h endpoint under control, GABA, Cd, and Cd + GABA treatments (*n* = 5 independent pots per treatment).

Parameter	Control	GABA	Cd	Cd+GABA
Shoot height (cm)	44.43 ± 1.06	43.85 ± 0.54	27.34 ± 0.31	37.73 ± 0.54
Root length (cm)	28.89 ± 0.55	29.87 ± 0.42	18.07 ± 0.46	23.95 ± 0.45
Leaf area (cm^2^)	357.7 ± 4.7	349.2 ± 4.2	222.1 ± 2.0	299.8 ± 2.6
Shoot dry weight (g)	3.04 ± 0.08	3.11 ± 0.04	1.89 ± 0.04	2.62 ± 0.04
Root dry weight (g)	1.35 ± 0.02	1.32 ± 0.02	0.84 ± 0.02	1.12 ± 0.00
Relative water content (%)	91.3 ± 0.9	90.3 ± 0.4	73.7 ± 0.4	88.8 ± 0.7
Electrolyte leakage (%)	11.9 ± 0.1	11.5 ± 0.3	36.8 ± 0.5	15.3 ± 0.4
Membrane stability index (%)	88.1 ± 0.1	88.5 ± 0.3	63.2 ± 0.5	85.5 ± 0.4
MDA (nmol g^−1^ FW)	4.37 ± 0.03	4.16 ± 0.07	9.86 ± 0.18	4.34 ± 0.09
Superoxide (nmol g^−1^ FW min^−1^)	0.62 ± 0.01	0.61 ± 0.00	1.65 ± 0.02	0.82 ± 0.01
Net photosynthesis (μmol CO₂ m^−2^ s^−1^)	20.48 ± 0.17	20.62 ± 0.39	12.58 ± 0.19	20.48 ± 0.24
Stomatal conductance (mol m^−2^ s^−1^)	0.390 ± 0.004	0.390 ± 0.009	0.238 ± 0.002	0.337 ± 0.006
F_v_/F_m_	0.827 ± 0.005	0.827 ± 0.001	0.724 ± 0.003	0.815 ± 0.003
ΦPSII	0.622 ± 0.003	0.621 ± 0.001	0.477 ± 0.002	0.576 ± 0.002
NPQ	0.761 ± 0.011	0.737 ± 0.006	1.230 ± 0.028	0.918 ± 0.013
Total chlorophyll (mg g^−1^ FW)	2.496 ± 0.006	2.485 ± 0.020	1.562 ± 0.016	2.107 ± 0.010
Carotenoids (mg g^−1^ FW)	0.422 ± 0.004	0.413 ± 0.005	0.256 ± 0.003	0.362 ± 0.008
SOD (U mg^−1^ protein)	41.10 ± 0.39	43.19 ± 0.59	66.96 ± 1.32	87.36 ± 1.30
CAT (μmol H₂O₂ min^−1^ mg^−1^ protein)	5.29 ± 0.06	5.39 ± 0.06	8.38 ± 0.08	11.18 ± 0.05
APX (μmol AsA min^−1^ mg^−1^ protein)	2.89 ± 0.05	2.96 ± 0.06	4.78 ± 0.06	6.55 ± 0.12
POD (U mg^−1^ protein)	6.80 ± 0.10	7.81 ± 0.16	11.25 ± 0.14	13.28 ± 0.20
GR (U mg^−1^ protein)	4.83 ± 0.06	5.02 ± 0.06	6.97 ± 0.22	8.77 ± 0.06
Ascorbate (μmol g^−1^ FW)	5.70 ± 0.10	5.88 ± 0.09	5.17 ± 0.06	6.36 ± 0.12
Glutathione (μmol g^−1^ FW)	2.44 ± 0.03	2.57 ± 0.06	2.89 ± 0.05	4.55 ± 0.06
Phytochelatins (μmol g^−1^ FW)	0.73 ± 0.01	0.84 ± 0.01	2.11 ± 0.04	3.32 ± 0.05
Total thiols (μmol g^−1^ FW)	3.36 ± 0.03	3.58 ± 0.05	4.76 ± 0.07	6.67 ± 0.09
Proline (μmol g^−1^ FW)	18.14 ± 0.39	18.99 ± 0.17	34.94 ± 0.80	41.03 ± 0.76
Soluble sugars (mg g^−1^ FW)	29.00 ± 0.34	31.89 ± 0.37	43.79 ± 0.74	51.26 ± 0.93
Glycine betaine (μmol g^−1^ DW)	5.82 ± 0.08	5.95 ± 0.08	10.30 ± 0.20	12.49 ± 0.26
Total phenolics (mg GAE g^−1^ DW)	18.51 ± 0.35	19.44 ± 0.22	22.77 ± 0.30	26.54 ± 0.28
Total flavonoids (mg QE g^−1^ DW)	10.79 ± 0.24	10.93 ± 0.24	13.21 ± 0.25	15.78 ± 0.49
Root Cd (mg kg^−1^ DW)	0.39 ± 0.01	0.41 ± 0.00	93.89 ± 2.54	61.72 ± 1.51
Shoot Cd (mg kg^−1^ DW)	0.16 ± 0.00	0.16 ± 0.00	44.51 ± 0.82	24.87 ± 0.94
Translocation factor	0.400 ± 0.007	0.381 ± 0.005	0.475 ± 0.015	0.403 ± 0.013
Root K (mg g^−1^ DW)	22.93 ± 0.18	22.81 ± 0.22	17.01 ± 0.37	21.17 ± 0.19
Root Ca (mg g^−1^ DW)	8.65 ± 0.12	8.76 ± 0.12	6.56 ± 0.11	8.22 ± 0.05
GABA response index	0.732 ± 0.030	1.142 ± 0.026	0.851 ± 0.032	1.252 ± 0.011

Values are the means ± standard error (SE). GABA, *γ*-aminobutyric acid; Cd, cadmium; FW, fresh weight; DW, dry weight; MDA, malondialdehyde; F_v_/F_m_, maximum quantum efficiency of photosystem II; ΦPSII, effective quantum yield of photosystem II; NPQ, non-photochemical quenching; SOD, superoxide dismutase; CAT, catalase; APX, ascorbate peroxidase; POD, peroxidase; GR, glutathione reductase; AsA, ascorbate; GAE, gallic acid equivalents; QE, quercetin equivalents. The table presents descriptive means; no significance letters appear in the supplied table, so differences between treatments should not be inferred from the displayed values alone. Percentages discussed in the text should be calculated from the displayed means, specifying the comparison group.

### 2.4. GABA concentration optimization assay

*γ*-Aminobutyric acid (≥99%, Sigma-Aldrich) was applied to the root zone at 0, 0.25, 0.5, 1.0, 2.0, and 5.0 mM in a preliminary screen at 50 mg Cd kg^−1^, in parallel with unstressed plants. The selected concentration had to maximize recovery under Cd without stimulating growth or photosynthesis in unstressed plants. This criterion did not assume complete biochemical inactivity; at 0.5 mM, unstressed plants later showed increases in the endogenous GABA response index and guaiacol peroxidase activity. Concentrations of 2.0 and 5.0  mM provided no additional benefit and slightly depressed root elongation compared to the control. Accordingly, 0.5  mM was selected, with 0.25 and 1.0  mM retained to document the dose-response.

### 2.5. Experimental design and pharmacological-dissection method

The main experiment comprised 11 treatments in a completely randomized design, with one plant per pot and five independently randomized pots per treatment (*n* = 55 experimental units). The pots were not divided among the treatments. For destructive sampling of signaling measurements, separate treatment-assigned pots were harvested at each sampling time. The regions of interest within a plant were considered technical subsamples and were averaged before analysis. Thus, the pot/plant mean, rather than an image region, was the unit of inference. Core treatments and pharmacological perturbations were applied as described below.

Pharmacological control and dose validation status. The reported 11-treatment matrix included Cd+GABA combinations with LaCl₃, EGTA, DPI, DMTU, or cPTIO, but no inhibitor-only arms (with or without Cd in the absence of GABA) and no rescue arm, including low-dose H₂O₂ after DPI or DMTU. An inhibitor-specific concentration series or phytotoxicity pre-screen was not documented in the available study record; therefore, nonphytotoxic doses cannot be claimed as established. The concentrations and application timing of these agents are not stated in the available manuscript and must be verified from the original protocol before reproducible reporting. The pharmacological comparisons assess the attenuation of a GABA-associated phenotype, not target specificity or pathway necessity.

### 2.6. Discrete-time sampling procedure

Signaling variables were measured on separate plants at 0, 30, 180, 360, and 1440 min after application. Because sampling was destructive, a single pot contributed to only one treatment-by-time cell, and there were no repeated observations from the same pot. The nominal 0-min sample was collected within 60 s after application and was not a true pre-treatment baseline. Five independent pot/plant means were analyzed per treatment-by-time cell; within-plant image regions were averaged and were not treated as independent replicates. The same unstressed Control 0-min mean (101.1 RFU for root Fluo-3 AM) was used as a cross-treatment for descriptive Ca^2+^-sensitive response amplitudes; it is not an individual plant baseline. Five sampled points cannot reconstruct onset, peak time, duration, or a continuous signaling signature.

### 2.7. Relative fluorescence responses, H₂O₂ and superoxide assays

Fluorescent probe approaches have been used to follow stress-induced cytosolic Ca^2+^ and redox changes in plants.^44-46^ The Ca^2+^-sensitive relative fluorescence response was measured in intact root tips (2-3 mm from the apex) and leaf mesophyll strips loaded with 10  μM Fluo-3 AM in loading buffer (10 mM MES, 5 mM KCl, 0.1 mM CaCl₂, pH 5.8) for 60 min at 4 °C, followed by a 30-min wash and 30 min of de-esterification at room temperature.^47^ Fluorescence was acquired at 488-nm excitation and 515-530-nm emission using identical microscope, detector, exposure, gain, and processing settings for all samples. Autofluorescence from matched unloaded tissue was subtracted. Five independently treated plants were imaged per treatment and time, and ten predefined regions of interest per plant were averaged to obtain one biological replicate value. Fluo-3 AM is non-ratiometric; therefore, RFU can be influenced by dye uptake, intracellular de-esterification, probe retention, tissue thickness, pH, and instrument response. No loading-uniformity dye, in situ minimum/maximum calibration, or conversion to absolute cytosolic Ca^2+^ concentration was performed. The values are consequently reported as Ca^2+^-sensitive relative fluorescence responses in RFU under standardized acquisition conditions, not Ca^2+^ concentrations or a calibrated Ca^2+^ signature. For descriptive summaries, the operational Ca^2+^-sensitive amplitude was calculated as the highest observed treatment mean among the five sampled points minus the unstressed Control 0-min root mean (101.1 RFU; Table 4). This shared post-application reference is not an individual pretreatment baseline. The tabulated time of the maximum identifies only the highest sampled observation. The previously reported Ca^2+^ AUC was withdrawn because its 100.4-RFU reference conflicted with Table 4, and complete treatment-by-time values for every pharmacological arm are not available for recalculation. Sparse, destructive sampling does not resolve continuous kinetics or a Ca^2+^ signature. The NO-associated relative fluorescence response was measured with 10  μM DAF-FM DA using the same loading, replication, acquisition control, background subtraction, and region-of-interest procedures (excitation 495 nm, emission 515 nm).^48^ DAF-FM DA is likewise non-ratiometric and is affected by loading, esterase activity, pH, retention, and reactive nitrogen chemistry; cPTIO sensitivity supports an NO-related component but does not make the signal quantitatively specific. NO results are presented as NO-associated relative DAF-FM fluorescence responses in RFU, not calibrated NO concentrations. The fixed 81.8-RFU operational reference used for reported NO amplitudes is close to, but distinct from, the displayed unstressed Control 0-min mean of 81.7 RFU (Table 4). Hydrogen peroxide was extracted in 0.1% (w/v) trichloroacetic acid and quantified from the reaction of the supernatant with potassium iodide in potassium phosphate buffer, with the absorbance read at 390 nm against an H₂O₂ standard curve.^49^ The superoxide production rate was determined from the hydroxylamine oxidation reaction, and the resulting nitrite being measured colorimetrically at 530 nm.^50^

**Table 4.** Exact values for Ca^2+^-sensitive relative fluorescence response, H₂O₂, and NO-associated relative fluorescence response in roots and leaves of *Salvadora persica* at 0, 30, 180, 360, and 1440 min after application. The nominal 0-min observation was collected within 60 s after application and was not a true pretreatment baseline. Fluorescence values are means ± standard error in RFU from five independent biological replicates and are not calibrated absolute concentrations.

Treatment	Time (min)	Root Ca^2+^-sensitive relative response (RFU)	Leaf Ca^2+^-sensitive relative response (RFU)	Root H₂O₂ (μmol g^−1^ FW)	Leaf H₂O₂ (μmol g^−1^ FW)	Root NO-associated relative response (RFU)	Leaf NO-associated relative response (RFU)
Control	0	101.1 ± 1.3	91.9 ± 1.1	2.07 ± 0.05	1.69 ± 0.02	81.7 ± 1.1	78.0 ± 1.2
Control	30	101.2 ± 2.0	94.3 ± 1.9	2.07 ± 0.04	1.73 ± 0.03	81.9 ± 1.3	77.7 ± 0.9
Control	180	98.5 ± 1.3	91.0 ± 2.1	2.10 ± 0.05	1.72 ± 0.03	82.0 ± 0.8	76.9 ± 1.3
Control	360	99.8 ± 1.5	91.3 ± 1.7	2.10 ± 0.03	1.74 ± 0.03	81.7 ± 2.5	78.2 ± 1.4
Control	1440	101.3 ± 1.5	91.0 ± 1.3	2.17 ± 0.03	1.69 ± 0.02	81.7 ± 0.9	76.9 ± 1.8
GABA	0	104.6 ± 2.0	95.6 ± 0.8	1.97 ± 0.02	1.58 ± 0.03	83.5 ± 0.8	79.6 ± 1.0
GABA	30	111.9 ± 1.1	98.2 ± 1.4	2.02 ± 0.03	1.67 ± 0.02	82.2 ± 1.5	78.6 ± 1.0
GABA	180	100.5 ± 0.8	90.2 ± 1.3	2.04 ± 0.05	1.69 ± 0.03	87.0 ± 1.7	77.9 ± 1.4
GABA	360	100.3 ± 1.7	93.6 ± 1.5	2.00 ± 0.05	1.65 ± 0.01	86.7 ± 2.4	83.4 ± 1.5
GABA	1440	98.3 ± 2.2	93.6 ± 1.1	1.98 ± 0.02	1.63 ± 0.04	81.1 ± 1.2	78.6 ± 1.3
Cd	0	134.6 ± 1.6	114.0 ± 2.0	5.15 ± 0.13	3.96 ± 0.06	85.6 ± 1.8	83.0 ± 0.4
Cd	30	153.8 ± 1.1	126.5 ± 0.9	5.29 ± 0.08	4.24 ± 0.07	86.6 ± 0.8	80.4 ± 0.7
Cd	180	96.5 ± 1.7	90.6 ± 1.5	6.01 ± 0.12	4.69 ± 0.04	101.2 ± 2.1	95.7 ± 0.8
Cd	360	99.5 ± 1.6	90.6 ± 0.7	5.14 ± 0.05	4.06 ± 0.05	120.5 ± 1.6	109.7 ± 0.8
Cd	1440	100.7 ± 0.8	89.9 ± 1.4	4.77 ± 0.12	3.78 ± 0.07	79.9 ± 2.3	76.8 ± 1.3
Cd+GABA	0	185.8 ± 3.6	155.8 ± 1.6	4.26 ± 0.04	3.26 ± 0.04	101.1 ± 1.6	93.3 ± 0.9
Cd+GABA	30	217.5 ± 1.1	187.4 ± 3.1	4.45 ± 0.06	3.55 ± 0.06	103.3 ± 1.9	96.5 ± 0.7
Cd+GABA	180	117.3 ± 1.8	103.3 ± 1.8	5.38 ± 0.07	4.40 ± 0.08	141.2 ± 2.2	126.1 ± 1.1
Cd+GABA	360	112.3 ± 2.3	102.9 ± 1.8	4.22 ± 0.10	3.27 ± 0.06	185.7 ± 3.4	157.2 ± 0.4
Cd+GABA	1440	113.3 ± 1.3	105.1 ± 0.8	3.78 ± 0.07	2.96 ± 0.04	94.8 ± 1.3	87.2 ± 1.3

The values were calculated directly from the replicate-level time-course dataset. This presentation reports the measured response magnitudes rather than the ANOVA F statistics.

### 2.8. Membrane damage and water-relation assays

Lipid peroxidation was estimated as MDA equivalents by the thiobarbituric acid reaction, correcting for non-specific absorbance (A₅₃₂–A₆₀₀) with an extinction coefficient of 155 mM^−1^ cm^−1^.^51^ Electrolyte leakage was measured as the ratio of the initial conductivity of leaf discs held in deionized water for 4 h at 25 °C to the conductivity after autoclaving, and the membrane stability index was calculated as 100 minus electrolyte leakage.^52^ Relative water content was determined on the youngest fully expanded leaf as (FW−DW)/(TW−DW) × 100, where TW is the turgid weight after 6 h of rehydration in the dark.^53^

### 2.9. Gas-exchange, chlorophyll-fluorescence and pigment assays

Net photosynthetic rate, stomatal conductance, transpiration rate, and intercellular CO₂ concentration were recorded between 09:00 and 11:00 h on the youngest fully expanded leaf using a portable infrared gas analyzer at a photosynthetic photon flux density of 1000  μmol m^−2^ s^−1^, a reference CO₂ concentration of 400  μmol mol^−1^, a leaf temperature of 28 °C, and 60% relative humidity. Chlorophyll fluorescence was measured on the same leaves after 30 min of dark adaptation; F_v_/F_m_ was calculated as (F_m_ - F_₀_)/F_m_ and the effective quantum yield of photosystem II (ΦPSII) and non-photochemical quenching (NPQ) were derived from light-adapted measurements according to standard definitions.^54^ ^,^ ^55^ Chlorophyll *a*, chlorophyll *b* and total carotenoids were extracted in 80% (v/v) acetone and calculated from absorbance at 663, 646 and 470 nm.^56^

### 2.10. Antioxidant-enzyme and non-enzymatic antioxidant assays

Fresh leaf tissue (0.5 g) was homogenized in ice-cold phosphate buffer and centrifuged as described. Soluble protein was quantified using the Bradford method.^57^ Superoxide dismutase, catalase, ascorbate peroxidase, guaiacol peroxidase, and glutathione reductase activities were determined using the specified spectrophotometric assays.^58-61^ Reduced ascorbate and GSH were quantified colorimetrically.^62^ ^,^ ^63^ Total phenolics were measured using the Folin‒Ciocalteu reagent,^64^ and total flavonoids using the aluminum chloride method.^65^ The spelling ‘guaiacol’ and the abbreviation GSH are used consistently throughout.

### 2.11. Osmolyte and endogenous GABA assays

Free proline was determined by the acid ninhydrin method at 520 nm,^66^ total soluble sugars by the phenol-sulfuric-acid method at 490 nm,^67^ and glycine betaine by the periodate method at 365 nm.^68^ Endogenous GABA was extracted in 70% (v/v) ethanol, purified on a cation-exchange column, and quantified against an authentic standard. The GABA response index (GRI) was the concentration in each plant divided by the mean concentration of unstressed controls; a GRI of 1.0 denotes the control level.

### 2.12. Cadmium, thiol-metabolism, and nutrient-element assays

Oven-dried root and shoot material (0.5 g) was digested in a 3:1 (v/v) HNO₃:HClO₄ mixture on a heating block until clear. Cd, K, Ca, Mg, Fe, and Zn were quantified by atomic absorption spectrophotometry against certified standards, with reagent blanks and a certified reference plant material included in every digestion batch. The bioconcentration factor was calculated as root Cd divided by substrate Cd, and the translocation factor was calculated as shoot Cd divided by root Cd.^69^ Non-protein thiols and total thiols were determined with 5,5′-dithiobis-(2-nitrobenzoic acid) at 412 nm,^70^ , and the phytochelatin pool was estimated as the difference between non-protein thiols and reduced glutathione.^71^

### 2.13. Growth, tolerance indices, and measured phenolic and flavonoid outcomes

At harvest, shoot height, root length, leaf area (by calibrated image analysis), fresh and dry biomass of shoots and roots, and the root-to-shoot ratio were recorded. The Cd tolerance index was calculated as the shoot dry weight expressed as a percentage of the mean unstressed control. The GABA recovery index was calculated as (X_t_ - XCd)/(XC - XCd) × 100, where X_t_, XCd, and XC are the shoot dry weights of the treatment, Cd alone, and unstressed control, respectively. Medicinally relevant chemistry was evaluated only through directly measured total phenolics (Folin-Ciocalteu; mg gallic acid equivalents g^−1^ DW) and total flavonoids (aluminum chloride; mg quercetin equivalents g^−1^ DW), using the procedures described in Section 2.10. A preliminary medicinal metabolite index combined control-normalized values with nominal equal weighting; however, it was not retained because the underlying total antioxidant capacity and benzyl isothiocyanate components, their replicate-level statistics, and a verifiable benzyl isothiocyanate analytical protocol were unavailable. Therefore, no benzyl isothiocyanate assay was performed in this study, and no compound-specific inferences were made.

### 2.14. Statistical analysis and multiplicity control

Each pot containing a single plant was considered as an experimental unit. For the four core groups (Cd absent/present × GABA absent/0.5 mM), Cd, GABA, and sampling time were specified as fixed effects with independent between-pot residual error; the remaining pharmacological treatments were compared as categorical treatment factors. A family of 37 endpoint omnibus tests was prespecified for Benjamini–Hochberg adjustment at q = 0.05, but the available tables report threshold *p*-values rather than the exact *p* and adjusted q outputs required to verify that gate and the subsequent Tukey groupings. Statistical analyses were performed using Statistix 8.1; correlations and PCA were generated in R,^72^ with NumPy, SciPy, and Matplotlib used for supporting computation and graphics.^73-75^ Analysis of variance and mean-comparison procedures followed standard biometrical methods.^76^ Probability thresholds printed in the available tables must not be represented as exact *p* or q values. No confirmatory path models were fitted.

Exact model specification. For the four core groups measured at each sampled time, Y_ijkr_ = μ+C_i_+G_j_+T_k_+(CG)_ij_+(CT)_ik_+(GT)_jk_+(CGT)_ijk_+ε_ijkr_, where C is Cd (0 or 50 mg kg^−1^), G is GABA (0 or 0.5 mM), T is the sampled time (0, 30, 180, 360, or 1440 min), and r denotes an independently assigned pot in that cell. Because each pot was harvested once, T is a between-pot factor and *ε* represents independent pot-to-pot variation; no repeated-measures covariance or pot random intercept is estimable. The nominal 0-min observation is post-application. This factorial model applies only to the four core groups, not to the 11-level pharmacological series.

For each 24-h endpoint across all 11 treatment groups, the appropriate one-way model is Y_ar_ = μ+A_a_+ε_ar_, with categorical treatment A_a_ (a = 1,…,11) and independent pot replicate r (normally five per group). The 11 groups did not form a complete Cd × GABA × inhibitor factorial design; the treatment model must not be described as a full three-way endpoint ANOVA. Time-course analyses of all 11 groups, if performed, require Y_akr_ = μ+A_a_+T_k_+(AT)_ak_+ε_akr_, with independent pots at each time. F-statistics, denominator degrees of freedom, and interaction *p*-values must be retrieved from the underlying analysis before numerical claims are made.

Assumptions and reporting status. Residual normality (for example, Shapiro–Wilk and Q–Q plots) and homogeneity of variance (for example, Levene or Brown–Forsythe tests and residual-versus-fitted plots) should be assessed separately for each fitted endpoint or time-course model. The supplied manuscript does not contain residuals or diagnostic results; therefore, neither assumption can be stated as passed. The tables give *p* < 0.001 for most outcomes and *p* = 0.525 for the root:shoot ratio; these are not exact *p* values for the former outcomes and do not provide the BH-adjusted q vector. Exact p/q values, omnibus η^2^, partial η^2^, and validated post-hoc letters require the original pot-level values and analysis output. No exact values have been inferred from rounded means and SEs.

Effect-size estimates are available from the displayed summaries. As transparent two-group descriptive estimates, Hedges g can be calculated from *n* = 5 per group and the displayed SEs using s_i_ = SE_i_√5, s_p_ = √[(s₁^2^+s₂^2^)/2], and g = [(meanCd+GABA − meanCd)/s_p_] × [1 − 3/(4(n_1_+n_2_−2)−1)]. The resulting approximate g is +7.37 for shoot dry weight (1.89 ± 0.04 versus 2.62 ± 0.04 g) and −15.67 for MDA (9.86 ± 0.18 versus 4.34 ± 0.09 nmol g^−1^ FW). These unusually large values depend on rounded summary SEs, describe only the Cd versus Cd+GABA contrasts, and are not ANOVA η^2^, pathway effects, or substitutes for pot-level inference.

## 3. Results

### 3.1. Cadmium stress impairs physiological performance in a dose-dependent manner

In the independent preliminary screening, Cd reduced *S. persica* shoot dry biomass by 9.8, 34.6, 48.3, 61.7, and 72.4% at 25, 50, 75, 100, and 150  mg Cd kg^−1^, respectively, relative to the screening control (3.06 g plant^−1^; Table 2). At 50  mg Cd kg^−1^, the mean was 2.00 g plant^−1^, and MDA increased by 120.9% (4.41–9.74 nmol g^−1^ FW). The root and shoot Cd concentrations increased linearly with applied Cd (R^2^ = 0.98 and 0.97). No mortality occurred up to 75  mg kg^−1^, whereas 100 and 150  mg kg^−1^ caused 12% and 28% mortality, respectively. These percentages refer only to the 21-d screening experiment; percentages for the subsequent main experiment were calculated from its separate treatment means.

### 3.2. 0.5 mM GABA provides the greatest recovery from cadmium stress

Root-applied GABA improved Cd-stressed plant performance at 0.25–1.0 mM, with the greatest recovery occurring at 0.5  mM (Figure 1). In unstressed plants, 0.5  mM GABA did not significantly alter shoot height, biomass, pigment content, or photosynthesis (*p* > 0.05), but it was not physiologically inert: the endogenous GABA response index increased by 56.0%, and guaiacol peroxidase activity increased by 14.9%. Thus, the lack of growth and photosynthetic effects should not be generalized to ‘no effect’; GABA produced selective biochemical responses even without Cd stress.

**Figure 1.**
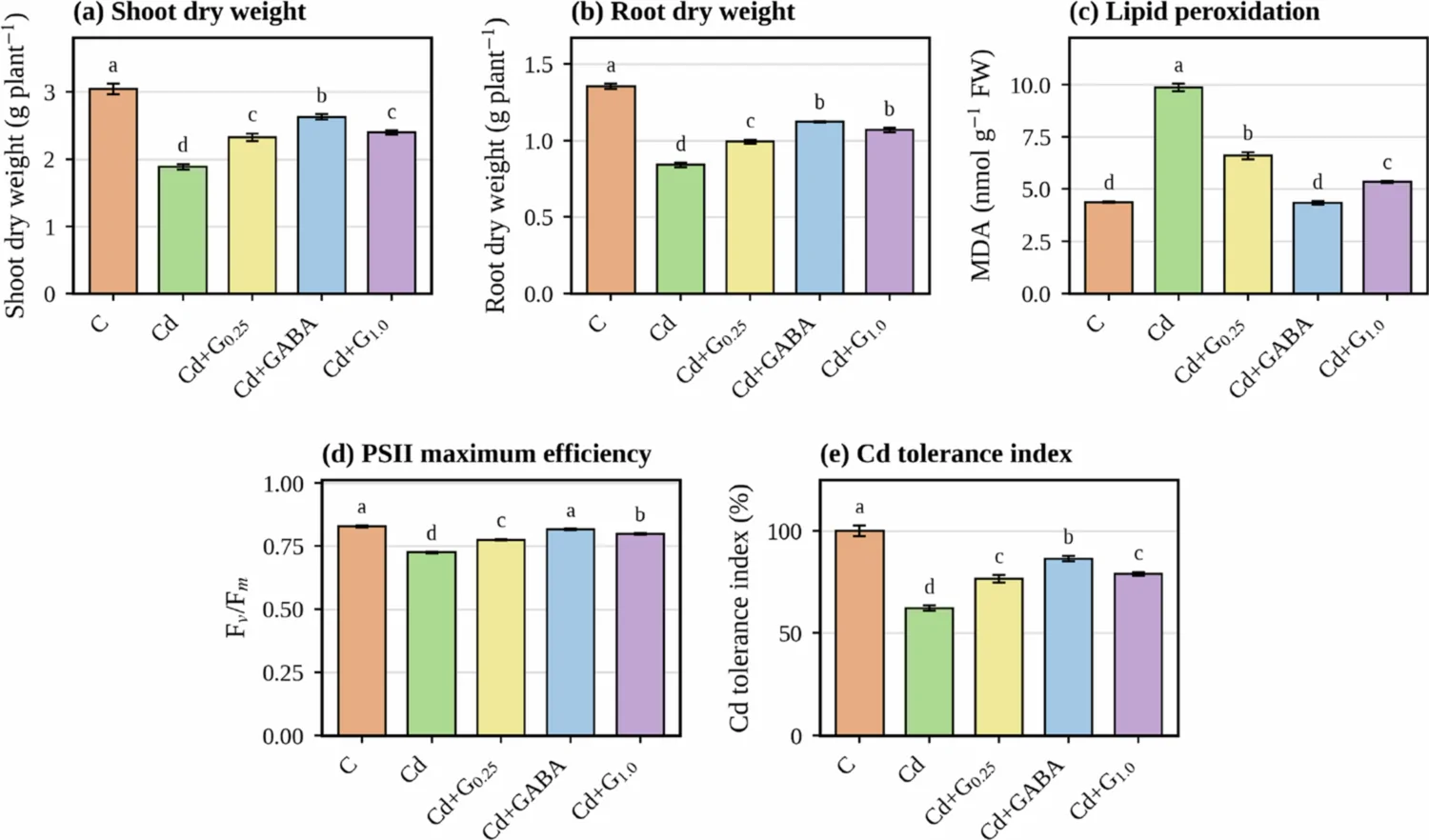
Effects of cadmium (Cd) and *γ*-aminobutyric acid (GABA) on (a) shoot dry weight, (b) root dry weight, (c) lipid peroxidation measured as malondialdehyde (MDA), (d) maximum quantum efficiency of photosystem II (*F*_v_/*F*_m_), and (e) Cd tolerance index in *Salvadora persica*. C, untreated control; Cd, Cd treatment; Cd+G₀.₂₅, Cd with 0.25 mM GABA; Cd+GABA, Cd with the study’s principal GABA dose; Cd+G₁.₀, Cd with 1.0  mM GABA. The bars show means with error bars. Different letters above the bars indicate statistically significant differences among the treatments.

### 3.3. GABA is associated with an altered Ca^2+^-sensitive relative fluorescence response

Among the five sampling times, the Ca^2+^-sensitive relative fluorescence response was highest at 30  min and approached the control range by 180  min (Figure 2a, b). In Cd-stressed roots, the observed maximum was 153.8 RFU at 30 min. Using the nominal 0-min unstressed-control value of 101.1 RFU as the operational reference, the operational amplitude was 52.7 RFU. With GABA, the 30-min value was 217.5 RFU and the operational amplitude was 116.4 RFU, 2.2-fold larger than that for Cd alone; the amplitude comparison was based on the corrected Control 0-min reference (Table 5). The leaves showed a comparable pattern of relative fluorescence response. GABA alone produced a smaller relative change (operational amplitude 10.8 RFU). These observations showed treatment-associated differences at the sampling times. They do not resolve sub-30-min kinetics, exact peak timing, dye-loading heterogeneity, or absolute cytosolic Ca^2+^ concentration, and therefore are described as a five-point fluorescence response profile rather than a reconstructed Ca^2+^ signature.

**Figure 2.**
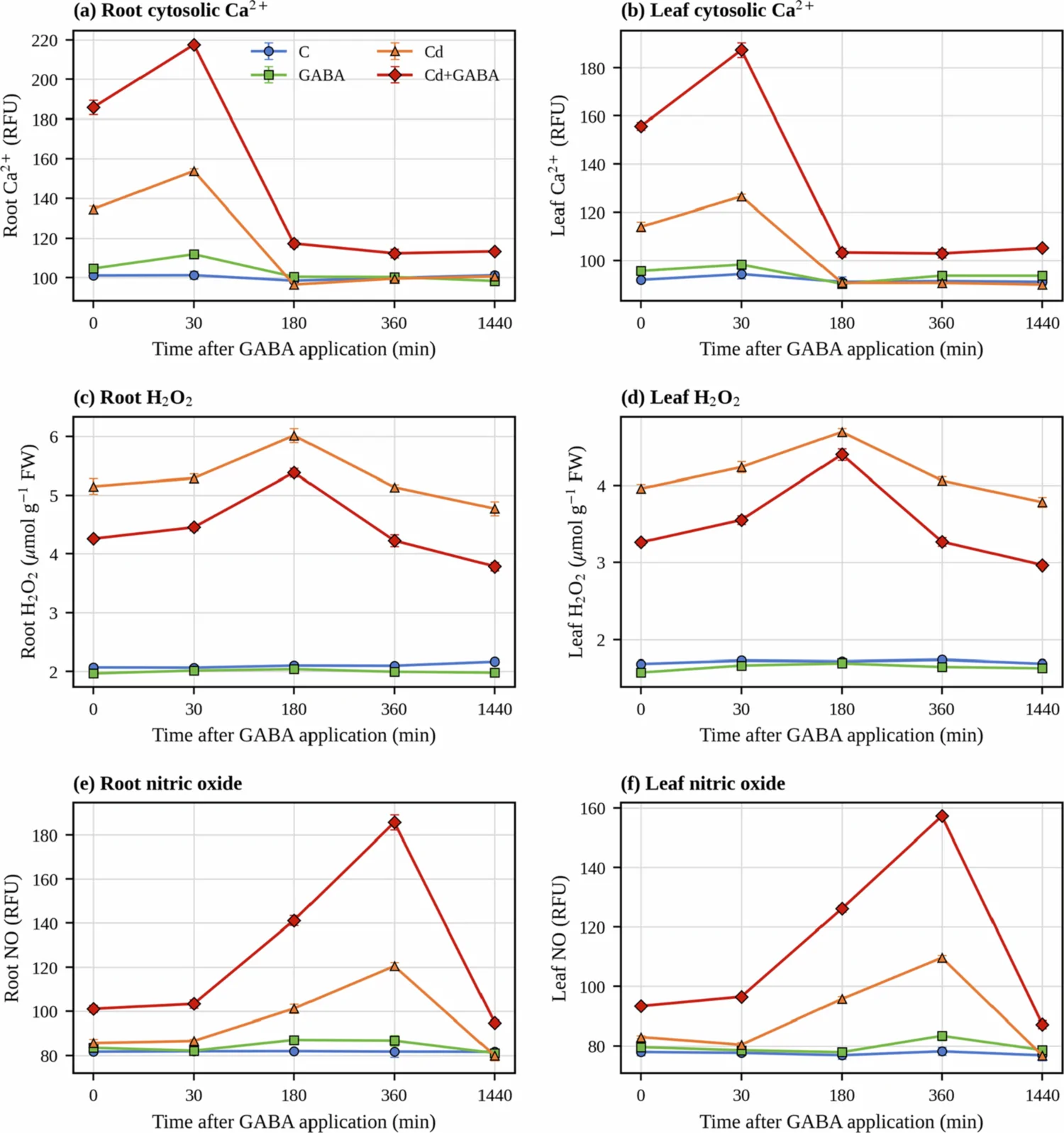
Relative fluorescence and H₂O₂ responses of *Salvadora persica* to Cd and GABA at five sampling times: Ca^2^+-sensitive Fluo-3 AM relative fluorescence response in (a) root tips and (b) leaf mesophyll, H₂O₂ in (c) roots and (d) leaves, and NO-associated DAF-FM DA relative fluorescence response in (e) roots and (f) leaves at 0, 30, 180, 360, and 1440  min after application. The treatments were: control (C), 0.5  mM GABA alone, 50 mg Cd kg^−1^ (Cd), and Cd+0.5  mM GABA. Symbols represent the mean ± standard error of five biological replicates; the x-axis is ordinal. The nominal 0-min sample was collected within 60 s after application and was not a true baseline. RFU values are relative, non-ratiometric signals and should not be interpreted as absolute Ca^2+^ or NO concentrations; the sparse sampling identifies observed maxima but not exact peak times or continuous kinetics.

**Table 5.** Descriptive indices derived from five sampled observations of root Ca^2+^-sensitive relative fluorescence response, and H₂O₂ and NO-associated relative fluorescence response. ‘Maximum’ is the highest observed treatment mean among the sampled times; operational Ca^2+^ amplitude is the maximum minus 101.1 RFU, whereas H₂O₂ and NO amplitudes use the stated fixed operational references; ‘time of maximum’ is the sampled time at which it occurred; and the reported H₂O₂ and NO AUC values are trapezoidal integrations against their fixed references in the uneven 0–1440-min intervals. The Ca^2+^ AUC is omitted because the prior reference is inconsistent with Table 4. However, these indices do not establish the exact peak timing or continuous kinetics.

Treatment	Ca^2+^ sampled maximum (RFU)	Ca^2+^ amplitude	Ca^2+^ time to peak (min)	Ca^2+^ AUC	H₂O₂ peak (μmol g^−1^ FW)	H₂O₂ amplitude	H₂O₂ time to peak (min)	H₂O₂ AUC	NO sampled maximum (RFU)	NO amplitude	NO time to peak (min)	NO AUC
C	101.3	0.2	1440	562	2.17	0.07	1440	36	82.0	0.1	180	35
GABA	111.9	10.8	30	1106	2.04	-0.06	180	0	87.0	5.2	180	4008
Cd	153.8	52.7	30	5470	6.01	3.91	180	4331	120.5	38.7	360	28057
Cd+G0.25	191.0	89.9	30	24384	5.76	3.66	180	3706	157.4	75.6	360	60146
Cd+GABA	217.5	116.4	30	29133	5.38	3.28	180	3032	185.7	103.8	360	84480
Cd+G1.0	204.2	103.1	30	22909	5.72	3.62	180	3475	168.4	86.6	360	68935
Cd+G+LaCl3	159.4	58.3	30	5856	5.71	3.61	180	3862	129.4	47.6	360	33958
Cd+G+EGTA	158.7	57.6	30	5795	5.83	3.73	180	3914	125.3	43.5	360	31439
Cd+G+DPI	166.8	65.7	30	6545	5.64	3.54	180	3715	132.8	51.0	360	36841
Cd+G+DMTU	158.9	57.8	30	5837	5.89	3.78	180	3732	134.8	53.0	360	37814
Cd+G+cPTIO	164.4	63.3	30	6264	5.68	3.58	180	3769	133.2	51.4	360	36670

Operational references were 101.1 RFU for Ca^2+^-sensitive relative fluorescence response, 2.10 μmol g^−1^ FW for H₂O₂, and 81.8 RFU for NO-associated relative fluorescence response. The Ca^2+^ reference is the displayed unstressed Control 0-min mean (Table 4), and the H₂O₂ and NO values are the fixed operational references used for their reported calculations and are close to their displayed Control 0-min means (2.07  μmol g^−1^ FW and 81.7 RFU). The nominal 0-min observation is a reference point rather than a true pre-treatment baseline. Ca^2+^ amplitudes were recalculated from 101.1 RFU. The previously reported Ca^2+^ AUC, computed against 100.4 RFU, has been withdrawn because full treatment-by-time means for all perturbation arms are not displayed for consistent recalculation.

### 3.4. GABA is associated with altered H₂O₂ and reduced oxidative injury

Hydrogen peroxide showed the highest sampled value at 180  min in both organs (Figure 2c, d). Cd raised root H₂O₂ to 6.01  μmol g^−1^ FW, whereas Cd+GABA reached 5.38  μmol g^−1^ FW, a 10.5% reduction. The descriptive 24-h H₂O₂ AUC decreased from 4331 to 3032 units (30.0%), and the 1440-min root value decreased from 4.27 to 3.38  μmol g^−1^ FW (20.8%). At 24 h, GABA reduced superoxide from 1.65 to 0.82 nmol g^−1^ FW min^−1^ (50.3%), MDA from 9.86 to 4.34 nmol g^−1^ FW (56.0%), and electrolyte leakage from 36.8% to 15.3% (58.4%) relative to Cd alone. The membrane stability index increased from 63.2% to 85.5%, a 35.3% relative increase. Each percentage was calculated directly from the means displayed in Tables 3–5.

### 3.5. NO-associated relative fluorescence differs at the sampled times

The NO-associated relative fluorescence response was highest at the 360-min sampling point in both roots and leaves (Figure 2e, f). In Cd-stressed roots, the observed maximum was 120.5 RFU; using the fixed operational reference of 81.8 RFU (close to the displayed unstressed 0-min mean of 81.7 RFU) gave a descriptive amplitude of 38.7 RFU. With GABA, the 360-min value was 185.7 RFU, and the operational amplitude was 103.9 RFU. Leaf NO-associated relative fluorescence showed a similar between-treatment pattern (operational amplitudes 79.7 versus 32.2 RFU). The observed maxima occurred at 30 min for Ca^2+^-sensitive relative fluorescence response, 180 min for H₂O₂, and 360 min for NO-associated relative fluorescence response; however, the five-point design cannot determine exact peak times, establish strict temporal ordering between unsampled intervals, or reconstruct continuous kinetics. At 24 h, residual root Ca^2+^-sensitive and NO-associated relative RFU values were positively correlated (r = 0.786, *p* < 0.001); this is an association between relative fluorescence measurements rather than evidence of shared upstream control.

### 3.6. Pharmacological treatments attenuate GABA-associated protection

Pharmacological perturbation attenuated GABA-associated responses (Figure 3). LaCl₃ and EGTA reduced the 30-min root Ca^2+^-sensitive relative fluorescence response from 217.5 to 159.4 and 158.6 RFU, and the 360-min NO-associated relative fluorescence response from 185.7 to 129.4 and 125.3 RFU, respectively. Relative to Cd+GABA shoot dry weight (2.62 g plant^−1^), the five perturbant means of 2.18–2.35 g plant^−1^ represented reductions of 10.3%–16.8%. Relative to Cd+GABA MDA (4.34 nmol g^−1^ FW), the perturbant means of 7.99–8.71 represented increases of 84.1%–100.7%. The GABA recovery index decreased from 64.0% to 25.2%–39.7% in the five treatments (Table 6). None reduced shoot dry weight below Cd alone. These calculations support the attenuation of GABA-associated protection but not target-specific causality. The fact that no perturbant group fell below Cd alone does not exclude phytotoxic or off-target effects relative to Cd+GABA; the design contains no inhibitor-only comparisons or rescue arms that would distinguish these explanations (Figures 4, 5, 6).

**Figure 3.**
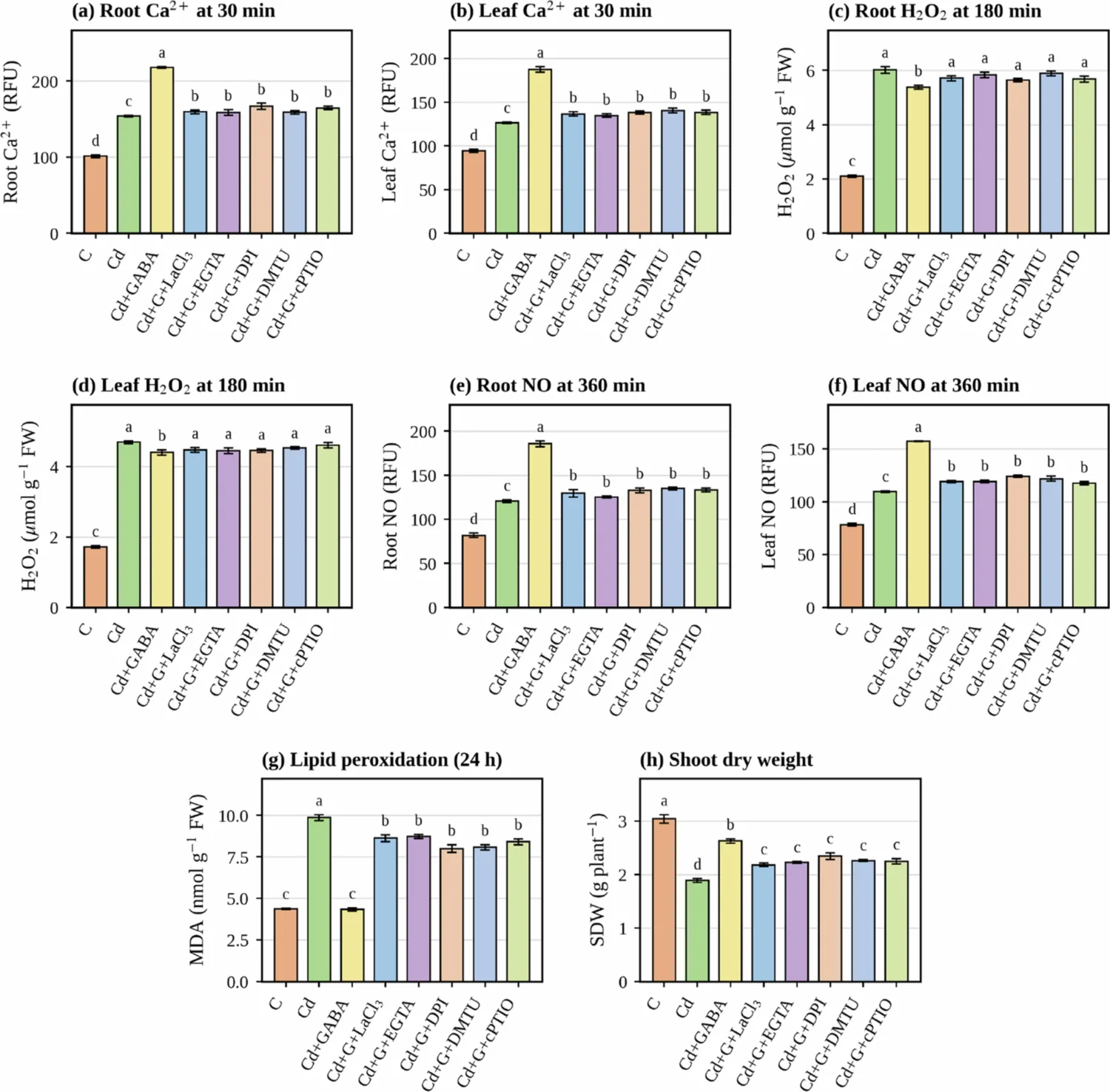
Effects of GABA and pharmacological treatments under Cd stress on (a) root and (b) leaf Ca^2+^ relative fluorescence responses at 30 min; (c) root and (d) leaf H₂O₂ at 180 min; (e) root and (f) leaf NO relative fluorescence responses at 360 min; (g) lipid peroxidation at 24 h; and (h) shoot dry weight. C, untreated control; GABA, *γ*-aminobutyric acid; LaCl₃, lanthanum chloride; EGTA, ethylene glycol tetraacetic acid; DPI, diphenyleneiodonium; DMTU, dimethylthiourea; cPTIO, nitric oxide scavenger. The bars represent treatment means with error bars. Different letters above the bars indicate the statistical groupings shown in the figure. The Ca^2+^ and NO values are relative fluorescence units (RFU), not calibrated concentrations. Because these inhibitors are not target-specific, their effects do not establish a linear or exclusively on-target Ca^2+^–ROS–NO pathway.

**Figure 4.**
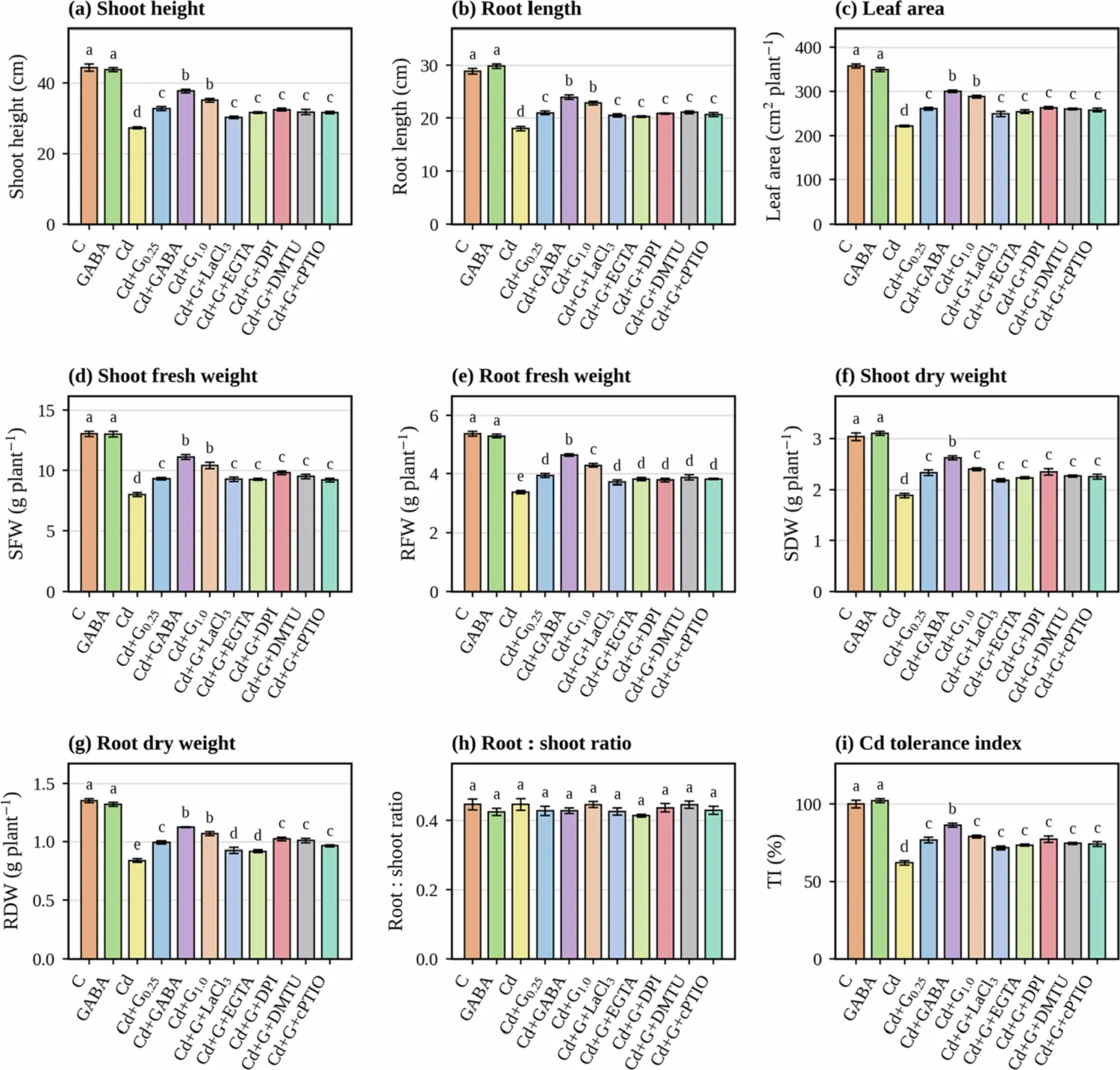
Growth responses of *Salvadora persica* 24 h after treatment: (a) shoot height, (b) root length, (c) leaf area, (d) shoot fresh weight, (e) root fresh weight, (f) shoot dry weight, (g) root dry weight, (h) root:shoot ratio, and (i) Cd tolerance index in all 11 treatments. The bars represent means ± standard error (*n* = 5); bars sharing a letter are not significantly different at *p* ≤ 0.05.

**Figure 5.**
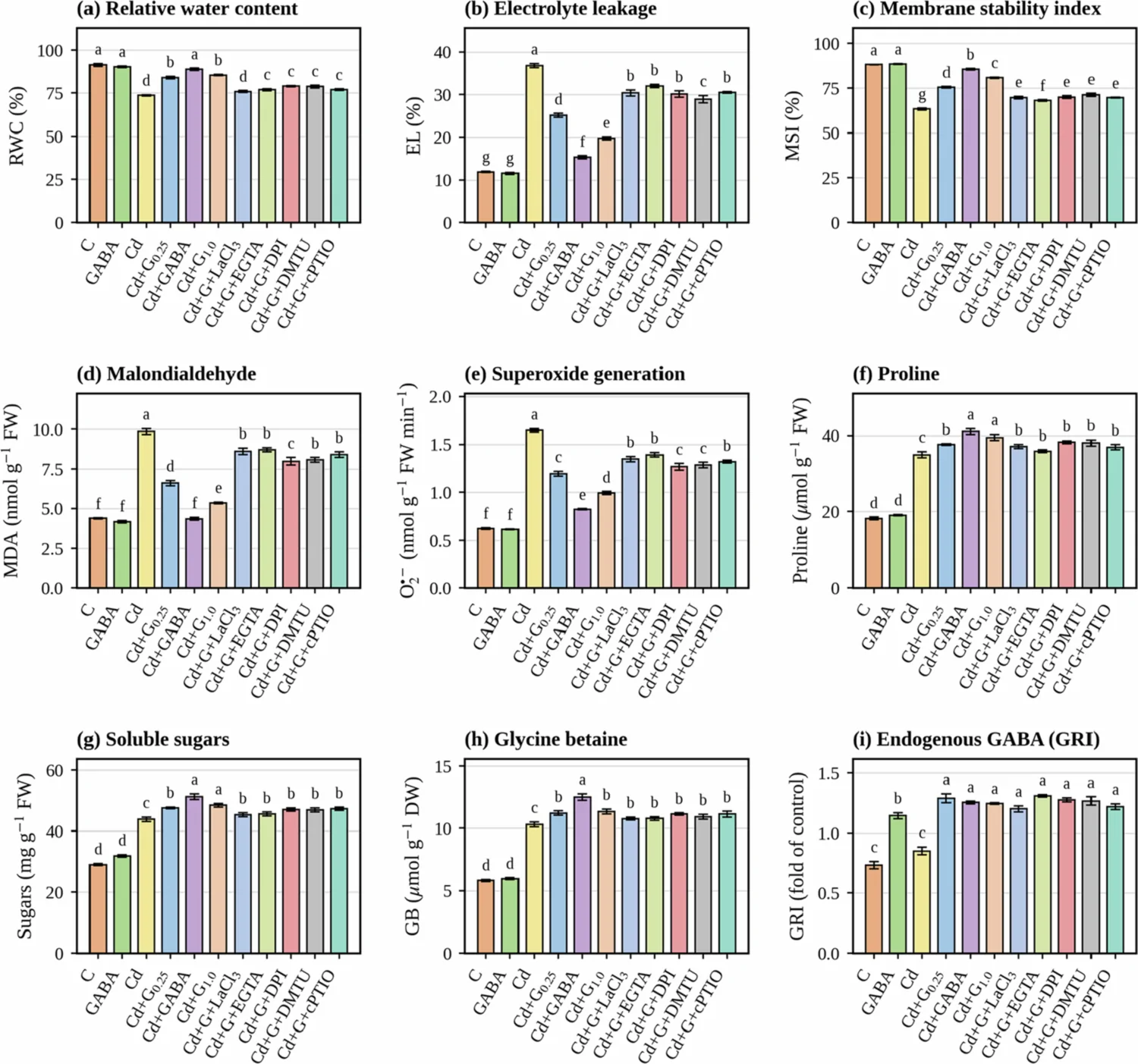
Water relations, membrane integrity, oxidative damage, and osmolytes: (a) relative water content, (b) electrolyte leakage, (c) membrane stability index, (d) malondialdehyde, (e) superoxide generation rate, (f) proline, (g) soluble sugars, (h) glycine betaine, and (i) the GABA response index (endogenous GABA relative to control). The bars are the means ± standard error (*n* = 5); bars sharing a letter are not significantly different at *p* ≤ 0.05.

**Figure 6.**
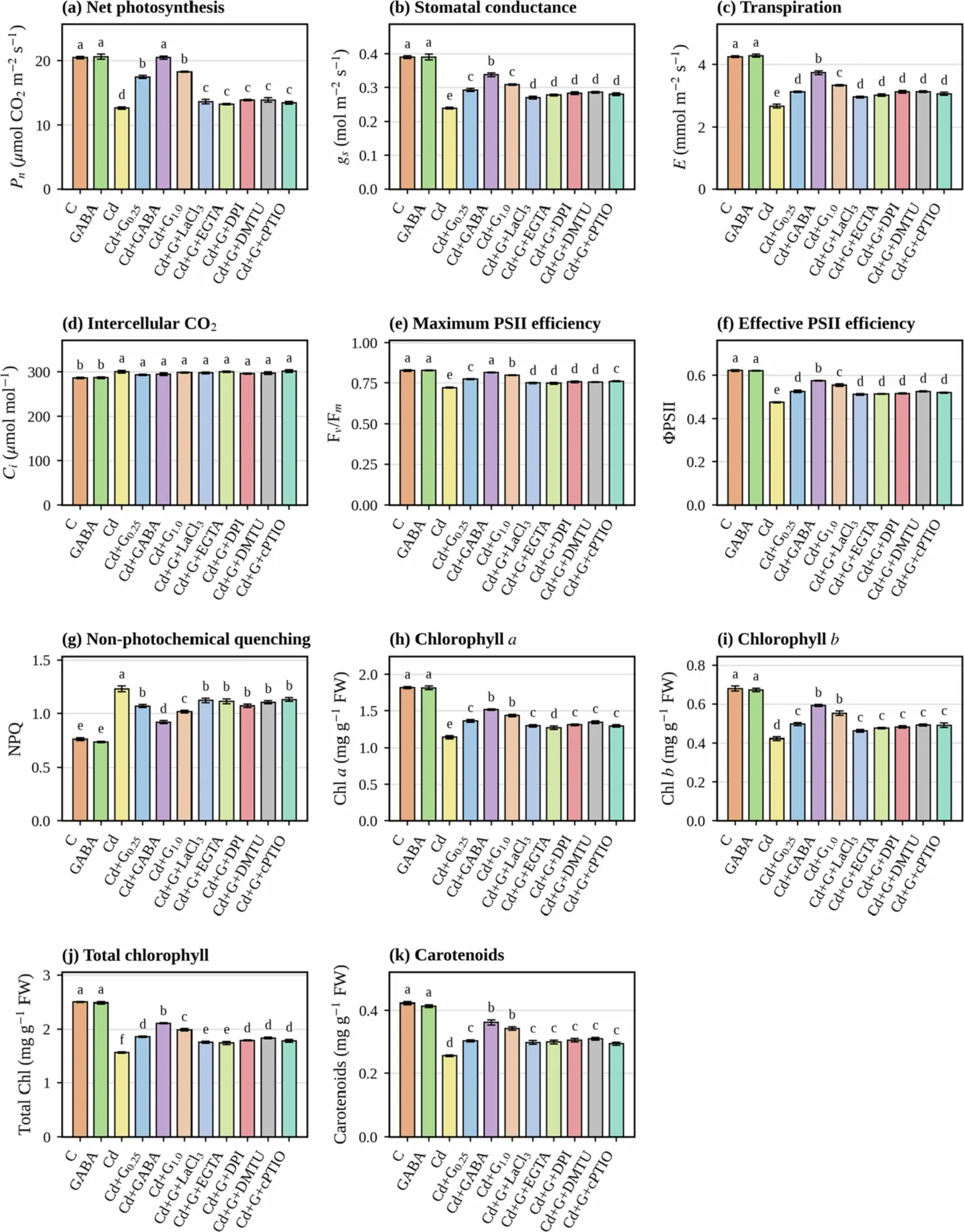
Photosynthetic and photochemical responses: (a) net photosynthetic rate, (b) stomatal conductance, (c) transpiration rate, (d) intercellular CO₂ concentration, (e) F_v_/F_m_, (f) ΦPSII, (g) non-photochemical quenching, (h) chlorophyll *a*, (i) chlorophyll *b*, (j) total chlorophyll, and (k) carotenoids. The bars are the means ± standard error (*n* = 5); the bars sharing a letter are not significantly different at *p* ≤ 0.05.

**Table 6.** Main experiment growth attributes, root:shoot ratio, Cd tolerance index, and GABA recovery index at 24 h. Values are means ± standard error from independent pots (*n* = 5). The final column contains the reported omnibus *p*-values or thresholds, not the verified adjusted q-values. Exact *p* and adjusted q values, omnibus effect sizes, and assumption diagnostics require the original pot-level analysis. Tukey letters are retained as originally reported and should be verified before manuscript submission.

Parameter	C	GABA	Cd	Cd+G₀.₂₅	Cd+GABA	Cd+G1.0	Cd+G+LaCl3	Cd+G+EGTA	Cd+G+DPI	Cd+G+DMTU	Cd+G+cPTIO	p
Shoot height (cm)	44.43 ± 1.06 a	43.85 ± 0.54 a	27.34 ± 0.31 d	32.69 ± 0.56 c	37.73 ± 0.54 b	35.09 ± 0.53 b	30.18 ± 0.32 c	31.58 ± 0.20 c	32.38 ± 0.40 c	31.72 ± 0.77 c	31.58 ± 0.37 c	<0.001
Root length (cm)	28.89 ± 0.55 a	29.87 ± 0.42 a	18.07 ± 0.46 d	20.97 ± 0.36 c	23.95 ± 0.45 b	22.81 ± 0.35 b	20.48 ± 0.28 c	20.22 ± 0.16 c	20.84 ± 0.12 c	21.06 ± 0.29 c	20.63 ± 0.42 c	<0.001
Leaf area (cm^2^ plant^−1^)	357.7 ± 4.7 a	349.2 ± 4.2 a	222.1 ± 2.0 d	260.2 ± 2.9 c	299.8 ± 2.6 b	287.5 ± 3.3 b	248.5 ± 6.0 c	253.3 ± 4.0 c	262.4 ± 3.0 c	259.9 ± 1.8 c	257.3 ± 4.0 c	<0.001
Shoot fresh weight (g plant^−1^)	13.03 ± 0.22 a	13.01 ± 0.24 a	8.01 ± 0.17 d	9.29 ± 0.11 c	11.10 ± 0.21 b	10.41 ± 0.27 b	9.24 ± 0.18 c	9.23 ± 0.09 c	9.78 ± 0.16 c	9.48 ± 0.17 c	9.19 ± 0.15 c	<0.001
Root fresh weight (g plant^−1^)	5.37 ± 0.08 a	5.29 ± 0.06 a	3.38 ± 0.06 e	3.93 ± 0.07 d	4.64 ± 0.04 b	4.28 ± 0.06 c	3.71 ± 0.07 d	3.81 ± 0.05 d	3.78 ± 0.06 d	3.87 ± 0.09 d	3.81 ± 0.02 d	<0.001
Shoot dry weight (g plant^−1^)	3.04 ± 0.08 a	3.11 ± 0.04 a	1.89 ± 0.04 d	2.33 ± 0.06 c	2.62 ± 0.04 b	2.40 ± 0.03 c	2.18 ± 0.03 c	2.23 ± 0.02 c	2.35 ± 0.06 c	2.26 ± 0.02 c	2.24 ± 0.05 c	<0.001
Root dry weight (g plant^−1^)	1.35 ± 0.02 a	1.32 ± 0.02 a	0.84 ± 0.02 e	0.99 ± 0.01 c	1.12 ± 0.00 b	1.07 ± 0.02 b	0.93 ± 0.02 d	0.92 ± 0.01 d	1.02 ± 0.01 c	1.01 ± 0.02 c	0.96 ± 0.01 c	<0.001
Root: shoot ratio	0.446 ± 0.016 a	0.425 ± 0.011 a	0.446 ± 0.017 a	0.428 ± 0.014 a	0.428 ± 0.008 a	0.446 ± 0.009 a	0.425 ± 0.011 a	0.413 ± 0.005 a	0.437 ± 0.012 a	0.446 ± 0.010 a	0.430 ± 0.012 a	0.525
Cd tolerance index (%)	100.0 ± 2.6 a	102.3 ± 1.3 a	62.1 ± 1.3 d	76.5 ± 1.9 c	86.3 ± 1.3 b	78.8 ± 1.0 c	71.6 ± 1.1 c	73.2 ± 0.6 c	77.1 ± 2.0 c	74.4 ± 0.6 c	73.8 ± 1.6 c	<0.001
GABA recovery index (%)	100.0 ± 6.8 a	106.1 ± 3.5 a	0.0 ± 3.5 d	38.0 ± 4.9 c	64.0 ± 3.5 b	44.2 ± 2.6 c	25.2 ± 2.9 c	29.4 ± 1.7 c	39.7 ± 5.4 c	32.5 ± 1.5 c	31.0 ± 4.2 c	<0.001

C, control; GABA, 0.5 mM GABA alone; Cd, 50 mg Cd kg^−1^; Cd+G₀.₂₅, Cd+GABA and Cd+G₁.₀, Cd with 0.25, 0.5 and 1.0 mM GABA; the remaining treatments combine Cd + 0.5 mM GABA with LaCl₃, EGTA, DPI, DMTU, or cPTIO.

### 3.7. GABA strengthens antioxidant and thiol-based defense under cadmium stress

Cd and GABA altered antioxidant and thiol metabolism (Figure 7). In unstressed plants, GABA increased the endogenous GABA response index by 56.0% (0.732 to 1.142) and guaiacol peroxidase activity by 14.9% (6.80 to 7.81 U mg^−1^ protein), while growth, pigment, and photosynthetic endpoints were unchanged. Under Cd stress, GABA increased GSH, phytochelatins, antioxidant activities, total phenolics, and total flavonoids relative to Cd alone. These findings support stress-enhanced action but do not support the broader claim that GABA has no effect in unstressed plants.

**Figure 7.**
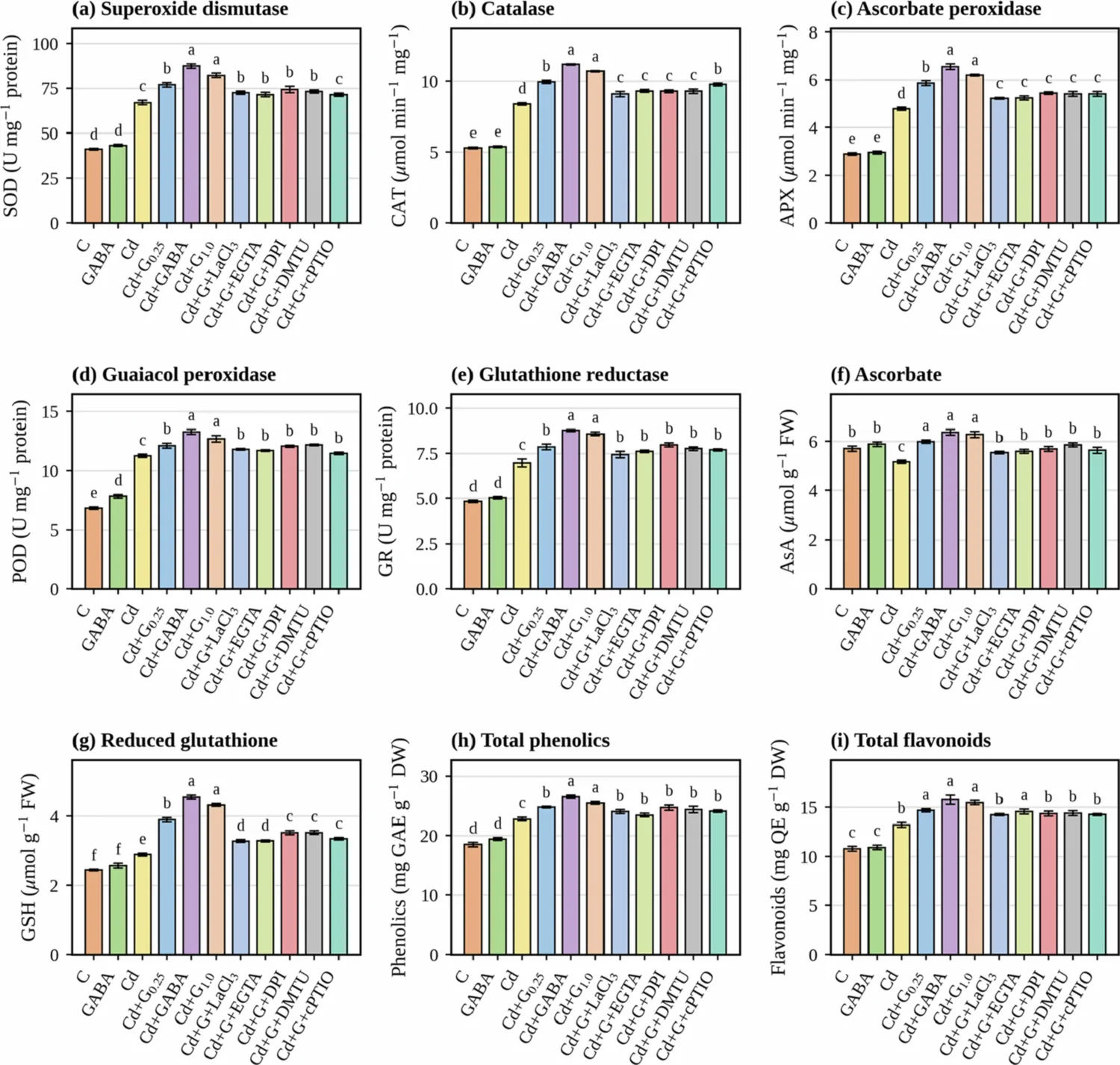
Enzymatic and non-enzymatic antioxidant responses: (a) superoxide dismutase, (b) catalase, (c) ascorbate peroxidase, (d) guaiacol peroxidase, (e) glutathione reductase, (f) ascorbate, (g) reduced glutathione, (h) total phenolics, and (i) total flavonoids. The bars are the means ± standard error (*n* = 5); the bars sharing a letter are not significantly different at *p* ≤ 0.05.

### 3.8. GABA reduces cadmium uptake, translocation and shoot loading

Under Cd stress, the root and shoot Cd concentrations were 93.89 and 44.51  mg kg^−1^ DW, respectively (Figure 8, Table 7). GABA reduced root Cd to 61.72 mg kg^−1^ DW (34.3%) and shoot Cd to 24.87 mg kg^−1^ DW (44.1%). The translocation factor declined from 0.475 to 0.403 (15.2%), and the bioconcentration factor decreased from 1.878 to 1.364 (27.4%). Relative to Cd alone, phytochelatins and total thiols increased by 57.3% and 40.1%, respectively. Cd reduced root K, Ca, Mg, Fe, and Zn by 25.8, 24.2, 23.6, 16.2, and 15.1%, respectively, compared to the control, and GABA increased these elements by 24.5, 25.3, 28.1, 16.0, and 19.0% compared to Cd alone. These percentages were calculated from the means in Tables 3 and 7.

**Figure 8.**
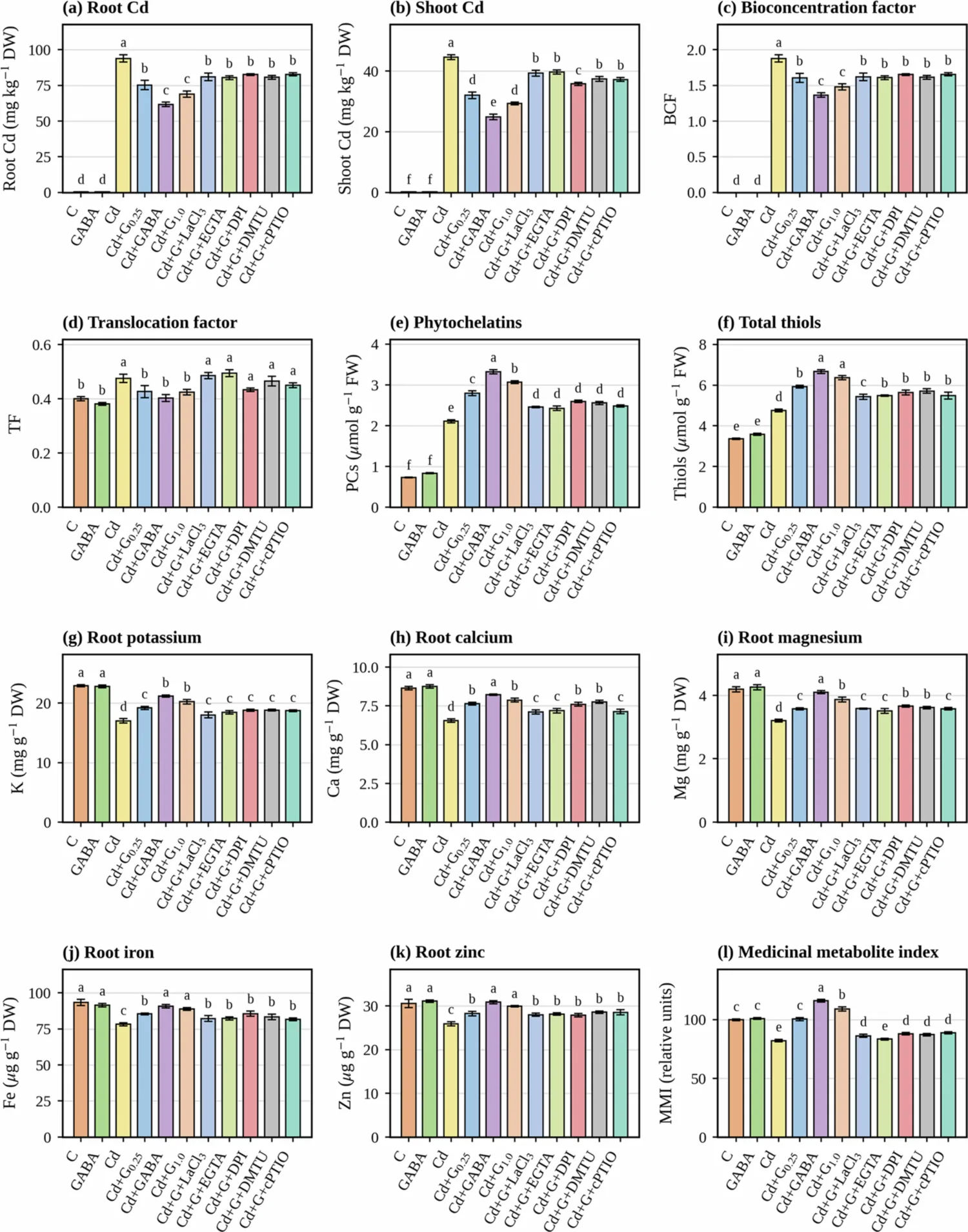
Cadmium accumulation, thiol-based detoxification, and cation nutrition. The medicinal metabolite index panel was removed from this figure. The bars represent the means ± standard error (*n* = 5); the letter groupings are reproduced from the source and require verification against the exact p/q output.

**Table 7.** Cadmium accumulation, translocation indices, thiol-based detoxification, and root cation concentration. Values are means ± standard error from independent pots (*n* = five). The final column contains reported omnibus *p*-values or thresholds; adjusted q-values and the Tukey gate require verification against pot-level output.

Parameter	C	GABA	Cd	Cd+G₀.₂₅	Cd+GABA	Cd+G1.0	Cd+G+LaCl3	Cd+G+EGTA	Cd+G+DPI	Cd+G+DMTU	Cd+G+cPTIO	p
Root Cd (mg kg^−1^ DW)	0.39 ± 0.01 d	0.41 ± 0.00 d	93.89 ± 2.54 a	75.27 ± 3.18 b	61.72 ± 1.51 c	68.86 ± 2.20 c	81.02 ± 2.66 b	80.42 ± 1.38 b	82.57 ± 0.70 b	80.64 ± 1.37 b	82.76 ± 1.18 b	<0.001
Shoot Cd (mg kg^−1^ DW)	0.16 ± 0.00 f	0.16 ± 0.00 f	44.51 ± 0.82 a	32.00 ± 1.07 d	24.87 ± 0.94 e	29.27 ± 0.43 d	39.26 ± 0.96 b	39.69 ± 0.69 b	35.76 ± 0.51 c	37.38 ± 0.83 b	37.20 ± 0.64 b	<0.001
Bioconcentration factor	0.000 ± 0.000 d	0.000 ± 0.000 d	1.878 ± 0.051 a	1.605 ± 0.064 b	1.364 ± 0.030 c	1.477 ± 0.044 c	1.620 ± 0.053 b	1.608 ± 0.028 b	1.651 ± 0.014 b	1.613 ± 0.027 b	1.655 ± 0.024 b	<0.001
Translocation factor	0.400 ± 0.007 b	0.381 ± 0.005 b	0.475 ± 0.015 a	0.426 ± 0.022 b	0.403 ± 0.013 b	0.425 ± 0.010 b	0.486 ± 0.011 a	0.494 ± 0.013 a	0.433 ± 0.006 a	0.465 ± 0.018 a	0.450 ± 0.009 a	<0.001
Phytochelatins (umol g^−1^ FW)	0.73 ± 0.01 f	0.84 ± 0.01 f	2.11 ± 0.04 e	2.79 ± 0.06 c	3.32 ± 0.05 a	3.07 ± 0.03 b	2.46 ± 0.02 d	2.43 ± 0.06 d	2.59 ± 0.03 d	2.56 ± 0.04 d	2.48 ± 0.02 d	<0.001
Total thiols (umol g^−1^ FW)	3.36 ± 0.03 e	3.58 ± 0.05 e	4.76 ± 0.07 d	5.93 ± 0.07 b	6.67 ± 0.09 a	6.36 ± 0.10 a	5.43 ± 0.12 c	5.48 ± 0.03 b	5.63 ± 0.12 b	5.71 ± 0.11 b	5.48 ± 0.16 b	<0.001
GSH (umol g^−1^ FW)	2.44 ± 0.03 f	2.57 ± 0.06 f	2.89 ± 0.05 e	3.89 ± 0.06 b	4.55 ± 0.06 a	4.32 ± 0.04 a	3.28 ± 0.04 d	3.29 ± 0.03 d	3.52 ± 0.06 c	3.52 ± 0.05 c	3.35 ± 0.03 c	<0.001
Root K (mg g^−1^ DW)	22.93 ± 0.18 a	22.81 ± 0.22 a	17.01 ± 0.37 d	19.19 ± 0.26 c	21.17 ± 0.19 b	20.22 ± 0.39 b	18.00 ± 0.48 c	18.45 ± 0.30 c	18.80 ± 0.17 c	18.82 ± 0.12 c	18.71 ± 0.15 c	<0.001
Root Ca (mg g^−1^ DW)	8.65 ± 0.12 a	8.76 ± 0.12 a	6.56 ± 0.11 d	7.64 ± 0.09 b	8.22 ± 0.05 a	7.87 ± 0.13 b	7.11 ± 0.14 c	7.19 ± 0.15 c	7.61 ± 0.12 b	7.76 ± 0.10 b	7.14 ± 0.15 c	<0.001
Root Mg (mg g^−1^ DW)	4.19 ± 0.08 a	4.26 ± 0.08 a	3.20 ± 0.04 d	3.57 ± 0.04 c	4.10 ± 0.05 a	3.87 ± 0.08 b	3.58 ± 0.02 c	3.51 ± 0.07 c	3.66 ± 0.03 b	3.61 ± 0.04 b	3.58 ± 0.04 c	<0.001
Root Fe (ug g^−1^ DW)	93.3 ± 2.2 a	91.6 ± 1.2 a	78.2 ± 1.1 c	85.4 ± 0.6 b	90.7 ± 1.3 a	88.8 ± 1.0 a	82.2 ± 2.0 b	82.3 ± 1.1 b	85.5 ± 1.8 b	83.3 ± 1.9 b	81.7 ± 0.9 b	<0.001
Root Zn (ug g^−1^ DW)	30.52 ± 0.95 a	31.06 ± 0.30 a	25.90 ± 0.49 c	28.22 ± 0.51 b	30.83 ± 0.35 a	29.91 ± 0.14 a	27.97 ± 0.34 b	28.11 ± 0.26 b	27.83 ± 0.40 b	28.54 ± 0.25 b	28.53 ± 0.57 b	<0.001

C, control; GABA, 0.5 mM GABA alone; Cd, 50 mg Cd kg^−1^; Cd+G₀.₂₅, Cd+GABA and Cd+G₁.₀, Cd with 0.25, 0.5 and 1.0 mM GABA; the remaining treatments combine Cd + 0.5 mM GABA with LaCl₃, EGTA, DPI, DMTU or cPTIO.

### 3.9. GABA improves photosynthetic measurements under cadmium stress

Relative to the control means in Tables 3 and 8, Cd reduced net photosynthesis by 38.6%, stomatal conductance by 39.0%, transpiration by 37.6%, F_v_/F_m_ by 12.5%, ΦPSII by 23.3%, total chlorophyll by 37.4%, and carotenoids by 39.3%, and intercellular CO₂ and NPQ increased by 4.9 and 61.6%, respectively. Relative to Cd alone, GABA increased net photosynthesis by 62.8%, ΦPSII by 20.8%, total chlorophyll by 34.9%, and carotenoids by 41.4%, whereas NPQ decreased by 25.4%. At the 24-h measurement, the net photosynthesis rate of the sampled leaf was 20.48  μmol CO₂ m^−^^2^ s^−1^ in both Cd+GABA and control plants; this single measurement does not establish sustained recovery of whole-plant carbon gain. The shoot dry weight was still 13.8% below that of the control (2.62 versus 3.04 g plant^−1^).

**Table 8.** Gas exchange, chlorophyll fluorescence, pigment content, water relations, and membrane damage traits. Values are means ± standard error from independent pots (*n* = 5 per treatment). The final column contains reported omnibus *p*-values or thresholds; adjusted q-values and Tukey gate require verification against pot-level output.

Parameter	C	GABA	Cd	Cd+G₀.₂₅	Cd+GABA	Cd+G1.0	Cd+G+LaCl3	Cd+G+EGTA	Cd+G+DPI	Cd+G+DMTU	Cd+G+cPTIO	p
Pn (umol CO_2_ m^−2^ s^−1^)	20.48 ± 0.17 a	20.62 ± 0.39 a	12.58 ± 0.19 d	17.45 ± 0.23 b	20.48 ± 0.24 a	18.26 ± 0.10 b	13.59 ± 0.33 c	13.19 ± 0.12 c	13.84 ± 0.09 c	13.85 ± 0.34 c	13.40 ± 0.22 c	<0.001
gs (mol m^−2^ s^−1^)	0.390 ± 0.004 a	0.390 ± 0.009 a	0.238 ± 0.002 e	0.292 ± 0.005 c	0.337 ± 0.006 b	0.309 ± 0.002 c	0.269 ± 0.004 d	0.277 ± 0.003 d	0.283 ± 0.004 d	0.286 ± 0.003 d	0.280 ± 0.004 d	<0.001
E (mmol m^−2^ s^−1^)	4.25 ± 0.03 a	4.29 ± 0.05 a	2.65 ± 0.06 e	3.12 ± 0.02 d	3.74 ± 0.06 b	3.33 ± 0.02 c	2.95 ± 0.03 d	3.01 ± 0.04 d	3.12 ± 0.05 d	3.12 ± 0.03 d	3.05 ± 0.05 d	<0.001
Ci (umol mol^−1^)	286.0 ± 1.8 b	286.6 ± 1.9 b	300.1 ± 3.0 a	293.0 ± 1.8 a	294.4 ± 2.9 a	298.1 ± 1.2 a	297.5 ± 1.9 a	300.1 ± 1.4 a	295.9 ± 1.0 a	296.7 ± 3.2 a	301.5 ± 2.9 a	<0.001
Fv/Fm	0.827 ± 0.005 a	0.827 ± 0.001 a	0.724 ± 0.003 e	0.774 ± 0.003 c	0.815 ± 0.003 a	0.798 ± 0.003 b	0.750 ± 0.002 d	0.748 ± 0.004 d	0.756 ± 0.005 d	0.756 ± 0.003 d	0.760 ± 0.004 c	<0.001
PhiPSII	0.622 ± 0.003 a	0.621 ± 0.001 a	0.477 ± 0.002 e	0.528 ± 0.006 d	0.576 ± 0.002 b	0.556 ± 0.005 c	0.513 ± 0.003 d	0.515 ± 0.002 d	0.518 ± 0.002 d	0.527 ± 0.002 d	0.522 ± 0.002 d	<0.001
NPQ	0.761 ± 0.011 e	0.737 ± 0.006 e	1.230 ± 0.028 a	1.069 ± 0.015 b	0.918 ± 0.013 d	1.016 ± 0.012 c	1.121 ± 0.020 b	1.113 ± 0.023 b	1.071 ± 0.015 b	1.104 ± 0.015 b	1.130 ± 0.018 b	<0.001
Total chlorophyll (mg g^−1^ FW)	2.50 ± 0.01 a	2.49 ± 0.02 a	1.56 ± 0.02 f	1.86 ± 0.01 d	2.11 ± 0.01 b	1.99 ± 0.02 c	1.75 ± 0.02 e	1.74 ± 0.03 e	1.79 ± 0.01 d	1.83 ± 0.02 d	1.78 ± 0.03 d	<0.001
Carotenoids (mg g^−1^ FW)	0.422 ± 0.004 a	0.413 ± 0.005 a	0.256 ± 0.003 d	0.303 ± 0.004 c	0.362 ± 0.008 b	0.342 ± 0.006 b	0.298 ± 0.006 c	0.299 ± 0.006 c	0.305 ± 0.005 c	0.310 ± 0.004 c	0.294 ± 0.005 c	<0.001
RWC (%)	91.33 ± 0.86 a	90.29 ± 0.43 a	73.65 ± 0.38 d	83.94 ± 0.68 b	88.76 ± 0.67 a	85.45 ± 0.42 b	75.88 ± 0.55 d	76.89 ± 0.53 c	79.01 ± 0.39 c	78.73 ± 0.76 c	76.92 ± 0.46 c	<0.001
Electrolyte leakage (%)	11.88 ± 0.13 g	11.51 ± 0.26 g	36.76 ± 0.51 a	25.09 ± 0.43 d	15.31 ± 0.36 f	19.72 ± 0.36 e	30.33 ± 0.70 b	31.97 ± 0.40 b	30.09 ± 0.74 b	28.88 ± 0.87 c	30.46 ± 0.21 b	<0.001
MDA (nmol g^−1^ FW)	4.37 ± 0.03 f	4.16 ± 0.07 f	9.86 ± 0.18 a	6.60 ± 0.17 d	4.34 ± 0.09 f	5.35 ± 0.06 e	8.62 ± 0.20 b	8.71 ± 0.12 b	7.99 ± 0.23 c	8.07 ± 0.15 b	8.40 ± 0.17 b	<0.001

C, control; GABA, 0.5 mM GABA alone; Cd, 50 mg Cd kg^−1^; Cd+G₀.₂₅, Cd+GABA and Cd+G₁.₀, Cd with 0.25, 0.5, and 1.0 mM GABA; the remaining treatments combined Cd + 0.5 mM GABA with LaCl₃, EGTA, DPI, DMTU, or cPTIO.

### 3.10. GABA improves growth and measures phenolic and flavonoid accumulation

In the main experiment, Cd reduced shoot height, root length, leaf area, shoot dry weight, and root dry weight by 38.5%, 37.5%, 37.9%, 37.8%, and 37.8%, respectively, relative to the corresponding control means (Tables 3 and 6). The resulting Cd tolerance index was 62.1%. GABA at 0.5  mM increased the shoot dry weight from 1.89 to 2.62 g plant^−1^ (38.6%), raised the Cd tolerance index to 86.3%, and produced a GABA recovery index of 64.0%. Total phenolics increased from 22.77 to 26.54  mg GAE g^−1^ DW (16.6%) and the total flavonoids from 13.21 to 15.78  mg QE g^−1^ DW (19.5%) relative to Cd alone; both outcomes differed significantly among the four core treatments (*p* < 0.001). The 37.8% main-experiment shoot-dry-weight reduction is distinct from the 34.6% value obtained in the independent preliminary screen. The approximate summary-derived Hedges g for the Cd+GABA versus Cd contrast was +7.37 for shoot dry weight and −15.67 for MDA (*n* = 5 per group; rounded means and SEs). These are descriptive two-group effect sizes, not verified omnibus η^2^.

### 3.11. Integrated analyses link GABA signaling with cadmium tolerance determinants

Tables 6–8 report omnibus *p* thresholds (mostly *p* < 0.001) and a reported *p* = 0.525 for root:shoot ratio. The exact omnibus *p-*values and 37 adjusted q-values are not available in this manuscript; therefore, the multiplicity-gated significance and displayed Tukey letters require verification against the pot-level analysis output. Pearson correlations and PCA used pooled treatment data (*n* = 55 independent endpoint pots) and were interpreted descriptively. Pooling the markedly separated treatment groups can inflate absolute correlations and dominate PCA components; these results do not estimate within-treatment relationships or causal effects. The Cd tolerance index was positively associated with ΦPSII (*r* = 0.949), total chlorophyll (0.945), stomatal conductance (0.939), carotenoids (0.925), and root dry weight (0.923) and negatively associated with shoot Cd (−0.940), superoxide (−0.929), root H₂O₂ (−0.926), root Cd (−0.921), and electrolyte leakage (−0.900). PCA summarized the covariance among standardized traits but did not estimate directed effects. Because a statistically identified path model with coefficients, standard errors, and fit statistics was unavailable, all previous path-analysis language was withdrawn.

## 4. Discussion

### 4.1. GABA-associated cadmium protection coincides with a Ca^2+^-ROS-NO response

The central question was whether GABA-mediated protection of *S. persica* from Cd is associated with the Ca^2+^-ROS-NO signaling module. Three observations support this association with the response network. First, GABA treatment was associated with a larger Ca^2+^-sensitive fluorescence response at 30 min, before the measured changes in antioxidant activity. Second, GABA alone produced a smaller Ca^2+^-sensitive fluorescence response and did not alter growth or photosynthesis at the measured endpoint, indicating a stronger observed response under Cd stress. Third, pharmacological perturbations attenuated the protective response; DPI, DMTU, and cPTIO also reduced the Ca^2+^-sensitive fluorescence peak, consistent with feedback or shared effects rather than a simple Ca^2+^ → ROS → NO hierarchy. Taken together, the concordant responses show pharmacological sensitivity of the GABA-associated phenotype, but are not specific tests of Ca^2+^, ROS, or NO mediation. They do not establish that each compound acted exclusively through its nominal target, that GABA directly initiated the module, or that the interactions form a strictly linear hierarchy.

This interpretation is consistent with the broader reappraisal of GABA as a signaling-active metabolite. GABA binding to the ALMT family and modulation of anion currents provide a plausible receptor-like mechanism in plants,^6^ ^,^ ^8^ and subsequent work has linked this interaction to stomatal regulation and water-use efficiency.^1^ Previous studies on GABA-mediated metal tolerance have mainly reported correlated changes in antioxidants and osmolytes.^2^ ^,^ ^3^ ^,^ ^7^ Our perturbation series demonstrates sensitivity to the tested compounds; however, its pharmacological nature cannot identify the direct molecular target of GABA, separate from on-target and off-target effects, or resolve the direction of every interaction within this feedback-linked network. No inhibitor-only controls were included under either unstressed or Cd-only conditions, and no low-dose H₂O₂ reversal of DPI/DMTU or other rescue experiment was performed in the reported treatment matrix. An inhibitor-specific dose-ranging or phytotoxicity pre-screen was not documented. Direct toxicity, nonspecific stress, and off-target actions cannot be separated from pathway-dependent attenuation.

### 4.2. GABA is associated with altered Ca^2+^-sensitive relative fluorescence

The five sampled times revealed a larger Ca^2+^-sensitive relative fluorescence response at 30 min in Cd+GABA plants and lower values at 180 min. This five-point pattern could reflect altered Ca^2+^-related probe fluorescence, but cannot resolve a Ca^2+^ signature or distinguish biological changes completely from probe-related variations. Fluo-3 AM is non-ratiometric, and without a loading-uniformity control or in situ calibration, the observed RFU differences may include contributions from dye loading, esterase activity, pH, retention, or tissue optical properties. Identical acquisition settings, unloaded-tissue background subtraction, independent biological replication, and averaged regions of interest reduced technical variability but did not eliminate these limitations. A Cd × GABA × time interaction was described in the source manuscript, but its F statistic and exact *p*-value are unavailable for verification; the displayed group means indicate differences at sampled times, while they do not determine the exact onset, duration, propagation, or absolute cytosolic Ca^2+^. Denser live imaging with ratiometric or genetically encoded Ca^2+^ reporters is required to establish those features.^13-15^ ^,^ ^47^

### 4.3. GABA preserves transient ROS signaling while limiting sustained oxidative damage

At the sampled 180-min point, root H₂O₂ was 10.5% lower in the Cd+GABA treatment than in the Cd alone treatment, while the descriptive 24-h AUC was 30.0% lower, and 24-h MDA was 56.0% lower. These calculations used the means shown in Tables 3–5. This pattern is compatible with the preservation of an early ROS response and the limitation of sustained oxidative damage, although sparse sampling and pharmacological non-specificity prevented a stronger causal inference. The reciprocal decrease in Ca^2+^-sensitive fluorescence under DPI and DMTU is consistent with feedback between Ca^2+^ and redox processes.^15^ ^,^ ^19-21^ ^,^ ^45^ The accompanying rise in enzymatic and non-enzymatic antioxidant capacity is consistent with the established role of these systems in limiting abiotic-stress-induced oxidative injury.^77^

### 4.4. NO-associated relative fluorescence within the response network

The sampled maxima for Ca^2+^-sensitive relative fluorescence response, H₂O₂, and NO-associated relative fluorescence response occurred at 30, 180, and 360 min, respectively. This spacing is compatible with temporally differentiated responses; however, five observations over 24 h cannot establish a strict sequential hierarchy or exclude earlier, later, or multiphasic events between samples. DAF-FM DA is non-ratiometric and responsive to aspects of reactive nitrogen chemistry; therefore, its RFU values are interpreted as NO-associated relative fluorescence responses rather than calibrated NO concentrations; loading, esterase activity, pH, and retention may also contribute to differences. Pharmacological sensitivity and the positive association between Ca^2+^- and NO-sensitive RFU are compatible with redox and NO-related associations; however, direct molecular and higher-frequency imaging experiments are required to determine directionality and targets.^22^ ^,^ ^23^ ^,^ ^25^

### 4.5. GABA-enhanced thiol metabolism restricts cadmium translocation and toxicity

GABA increased glutathione by 57.4%, phytochelatins by 57.3%, and total thiols by 40.1% relative to Cd alone (Tables 3 and 7). Phytochelatins were correlated with glutathione (r = 0.903) and root Cd (r = 0.782). Shoot Cd decreased by 44.1%, root Cd decreased by 34.3%, and the translocation factor decreased by 15.2%. These table-verified changes are consistent with reduced uptake and proportionally greater protection of aerial tissues, while the pharmacological design does not identify the molecular step responsible.^33-35^ ^,^ ^71^ Restricted root-to-shoot transfer of this kind is also central to the phytostabilization behavior described for metal-exposed species.^78^

### 4.6. GABA-associated responses coincide with improved photosynthetic performance

Cd inhibits photosynthesis through both stomatal and non-stomatal limitations. At the 24-h leaf gas-exchange measurement (09:00–11:00  h), net photosynthesis in Cd+GABA plants equaled the control mean (20.48 μmol CO₂ m^−2^ s^−1^), while F_v_/F_m_ increased from 0.724 under Cd alone to 0.815 with GABA, and NPQ decreased from 1.230 to 0.918. This instantaneous leaf-level rate cannot establish whether daily whole-plant carbon assimilation or earlier photosynthesis had returned to control values. The shoot dry weight remained 13.8% below that of the control (2.62 versus 3.04 g plant^−1^). Relative to controls, Cd+GABA plants still had 16.2% less leaf area (299.8 versus 357.7 cm^2^), 17.0% lower root dry weight (1.12 versus 1.35 g), and 17.1% shorter roots (23.95 versus 28.89 cm). The root K and Ca also remained below the control (21.17 versus 22.93 mg g^−1^ DW and 8.22 versus 8.65 mg g^−1^ DW, respectively), the relative water content was 88.8 versus 91.3%, and Cd persisted in the roots and shoots (61.72 and 24.87 mg kg^−1^ DW, respectively). These residual limitations, together with growth loss before the measurement, could explain why the biomass did not fully recover despite a control-level leaf photosynthesis reading being recorded. The present endpoint data do not distinguish between their individual contributions or establish a causal explanation. The protection of the photosynthetic apparatus on this scale is consistent with the established link between antioxidant capacity and maintenance of chloroplast function under abiotic stress.^79^ All percentage comparisons in this interpretation used the displayed means in Tables 3, 6, and 8.

### 4.7. GABA increases measured total phenolics and flavonoids during cadmium stress

For medicinal species, growth tolerance alone does not determine product quality. Compared to Cd alone, Cd+GABA increased the total phenolics by 16.6% (22.77–26.54  mg GAE g^−1^ DW) and total flavonoids by 19.5% (13.21–15.78  mg QE g^−1^ DW); both table-supported comparisons were significant. These assays describe aggregate chemical classes rather than individual compounds. The medicinal metabolite index and benzyl-isothiocyanate claims were withdrawn because their component-level evidence and analytical methods could not be verified (Figures 9 and 10).

**Figure 9.**
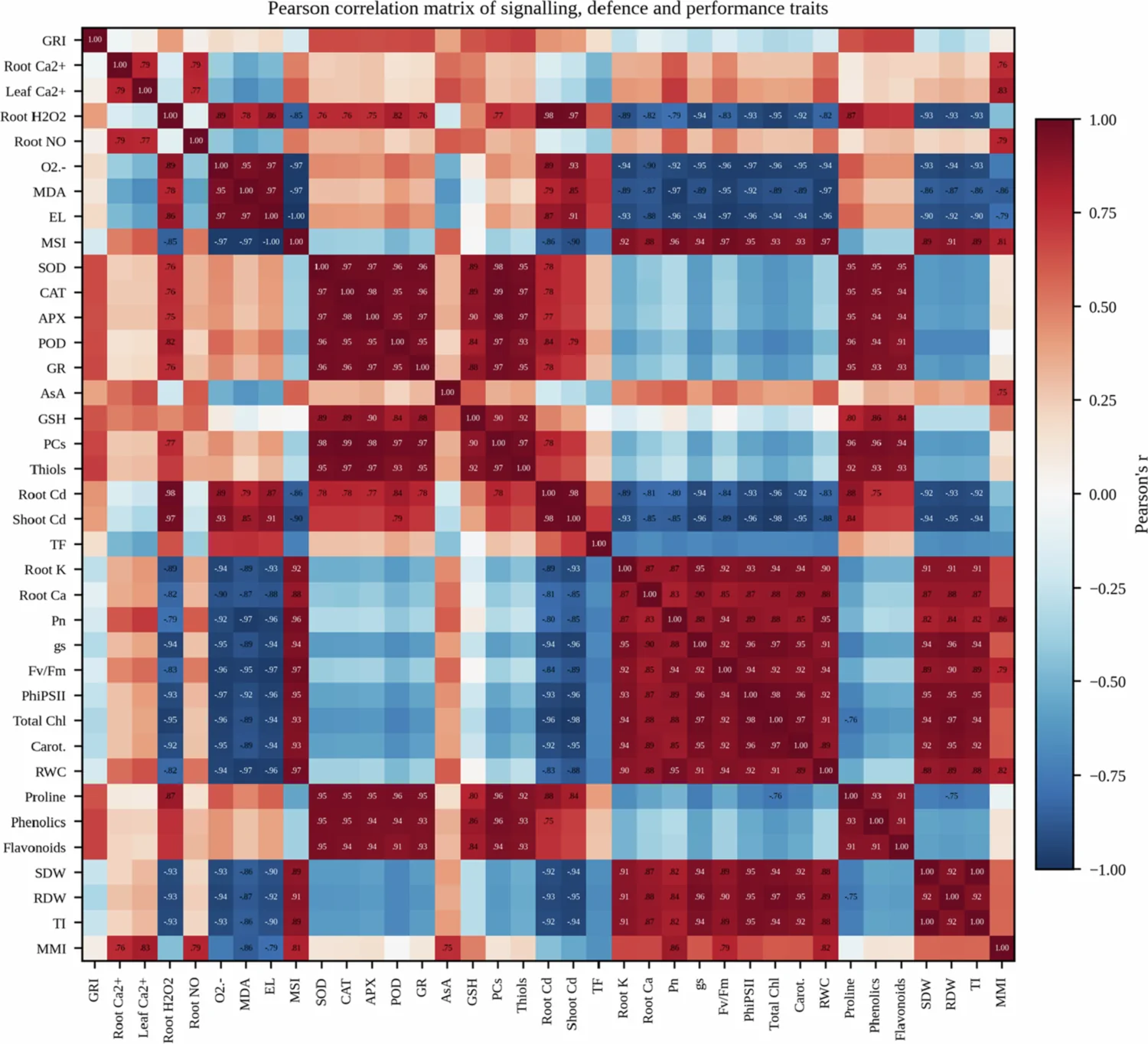
Exploratory Pearson correlation matrix of traits pooled across treatments (*n* = 55 independent endpoint pots). Between-treatment differences can inflate coefficients and do not establish within-treatment correlations. Coefficients describe associations and are not path coefficients or evidence of directionality. Multiplicity-controlled treatment comparisons are reported in Tables 6–8.

**Figure 10.**
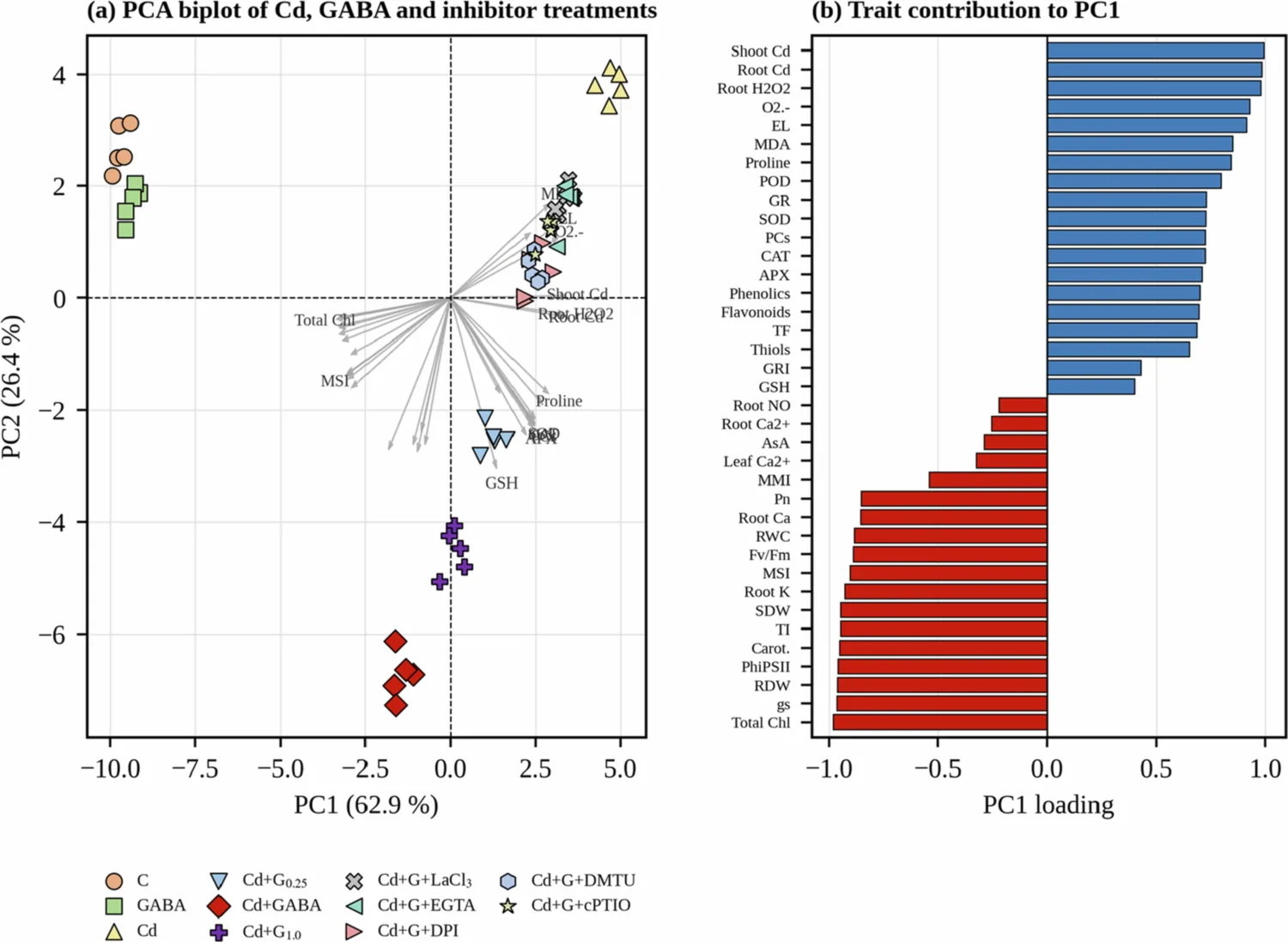
Exploratory PCA of standardized endpoint traits pooled across treatments (*n* = 55): (a) scores and loadings biplot and (b) contributions to PC1. Treatment separation may dominate PCA scores/loadings; PCA does not estimate within-treatment associations, directed paths, indirect effects, or causal coefficients.

### 4.8. Limitations of pharmacological inference

This study has several statistical limitations. With five independent pots per treatment or treatment-by-time cell, interaction estimates have limited precision, and residual diagnostics have modest power. Benjamini–Hochberg adjustment was specified for the family of 37 endpoint tests, but adjusted q-values and exact input *p*-values are absent from the available output; the asserted q < 0.05 gate and Tukey letters cannot be independently verified. Assumption-check results for residual normality and equal variance are also unavailable; a small *n* further limits diagnostic power. Pooled-group correlations and PCA remain exploratory because treatment separation may induce or amplify associations. Figure 11 is a conceptual hypothesis, not a fitted path model. No confirmatory path analysis was performed; therefore, it provides neither path coefficients nor global fit indices; it should be tested in an independent, larger dataset using a prespecified identifiable model and complete reporting of standardized coefficients, confidence intervals, R^2^ values, and fit measures. Although shoot dry weight with each perturbant remained above Cd alone, this comparison does not establish the absence of inhibitor toxicity: the relevant inhibitor-only, Cd+inhibitor, and rescue controls are missing. Dose, application timing, and phytotoxicity screening for the pharmacological agents also require confirmation in the experimental record before the treatments can be fully reproduced. The pharmacological results should be interpreted as treatment-associated attenuation rather than proof of a required molecular pathway.

**Figure 11.**
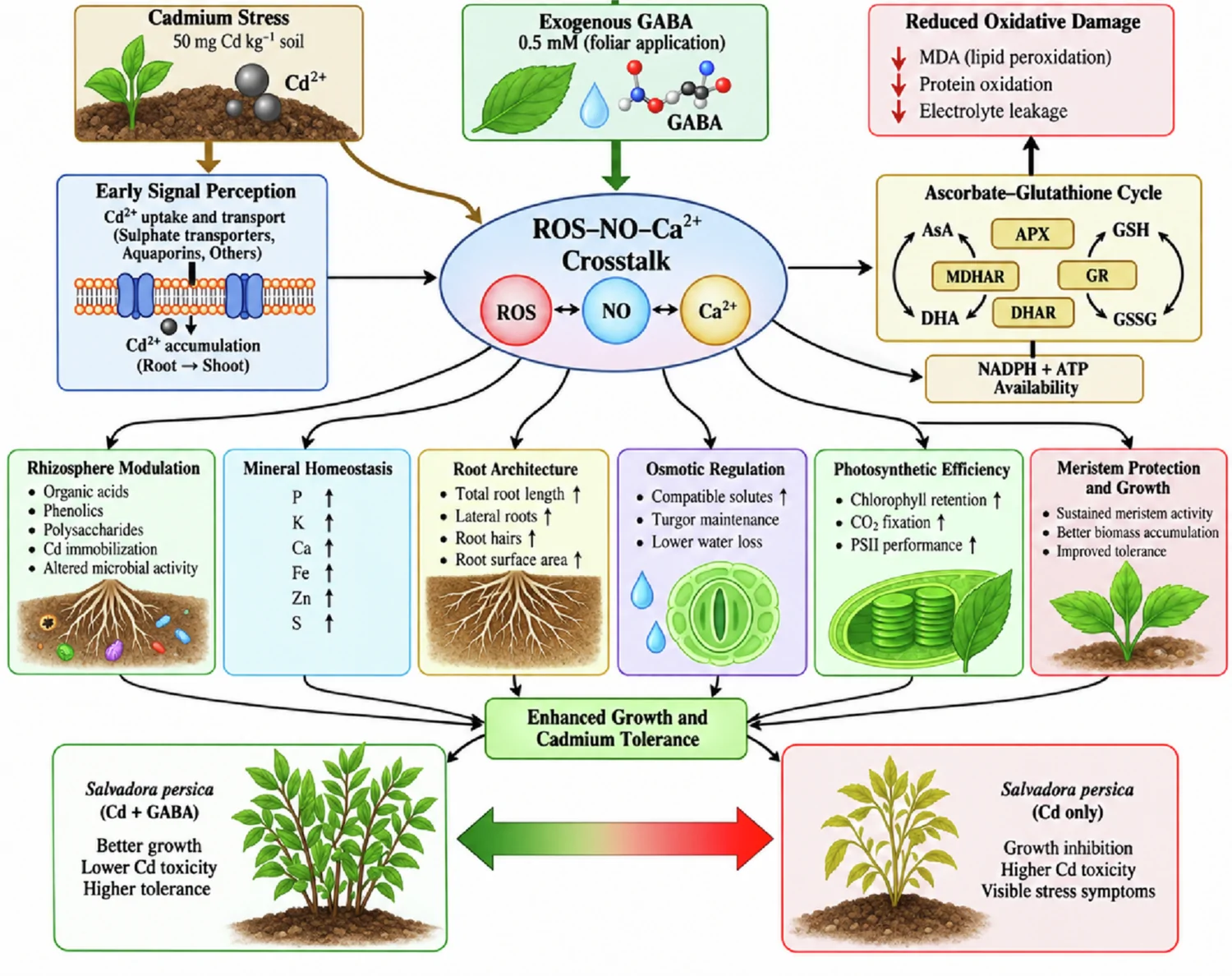
Conceptual evidence map of GABA-associated Cd tolerance in *Salvadora persica*. Exogenous GABA is associated with ROS-NO-Ca^2+^ signaling, antioxidant defense, reduced oxidative damage, improved mineral and water balance, root growth, photosynthetic performance, and enhanced Cd tolerance in plants. The arrows indicate proposed associations and do not establish causal direction or fitted path coefficients.

## 5. Conclusion

Exogenous GABA mitigated cadmium toxicity in *Salvadora persica* in association with differences in Ca^2+^-sensitive and NO-associated relative fluorescence responses and H₂O₂ at the five sampled times. Treatment effects on the method-supported endpoint outcomes were evaluated with pot/plant as the experimental unit and specified in a 37-outcome Benjamini–Hochberg family; exact adjusted q-values and residual diagnostics are unavailable for verification. The observed responses coincided with stronger thiol/redox defenses, reduced Cd accumulation and translocation, improved leaf photosynthetic measurements and growth at the measured endpoint, and higher total phenolic and flavonoid concentrations. The correlation and PCA results describe multivariate associations only. The attenuation by LaCl₃, EGTA, DPI, DMTU, and cPTIO shows the sensitivity of the GABA-associated phenotype to the tested compounds, while the effects of DPI, DMTU, and cPTIO on the Ca^2+^-sensitive peak suggest feedback. These interventions do not establish that GABA acts upstream, through a linear pathway, or compound-specific effects. Figure 11 presents a conceptual hypothesis, not a fitted path model. Without documented inhibitor phytotoxicity screening, inhibitor-only controls, or a rescue experiment, these findings cannot distinguish pathway perturbation from compound toxicity or off-target effects.

### 5.1. Future research directions

Future studies should use larger independent samples and a preregistered analysis plan that defines the primary outcomes and multiplicity families. A confirmatory path or structural equation model should be specified before analysis and reported with standardized path coefficients, standard errors, or confidence intervals, direct and indirect effects, endogenous variable R^2^, identification checks, and appropriate global fit statistics such as χ^2^, CFI, TLI, RMSEA with confidence interval, and SRMR. However, independent validation data, dense live imaging, genetic perturbation, and controlled rescue experiments are required before causal interpretations can be made.

## Supplementary Material

Supplementary Table S2 Figure10 PC1.csv Supplementary Table S2 Figure10 PC1.csv

Supplementary Material Supplementary TableS1 Figure 9 Pearson_Correlation_Matrix.csv

## Data Availability

The datasets generated and analyzed during the current study are included in the supplementary material. The data underlying Figures 9 and 10 are provided in Supplementary Tables S1 and S2, respectively. Additional supporting data are available from the corresponding author upon reasonable request.

## Statement on guidelines

All experimental studies and materials involved in this research comply with relevant institutional, national, and international guidelines and legislation.
